# Unphosphorylated SR-Like Protein Npl3 Stimulates RNA Polymerase II Elongation

**DOI:** 10.1371/journal.pone.0003273

**Published:** 2008-09-26

**Authors:** Jessica L. Dermody, Jonathan M. Dreyfuss, Judit Villén, Babatunde Ogundipe, Steven P. Gygi, Peter J. Park, Alfred S. Ponticelli, Claire L. Moore, Stephen Buratowski, Miriam E. Bucheli

**Affiliations:** 1 Department of Biological Chemistry and Molecular Pharmacology, Harvard Medical School, Boston, Massachusetts, United States of America; 2 Department of Molecular Biology and Microbiology, Tufts University School of Medicine, Boston, Massachusetts, United States of America; 3 Department of Cell Biology, Harvard Medical School, Boston, Massachusetts, United States of America; 4 Department of Biochemistry, School of Medicine and Biomedical Sciences, State University of New York, Buffalo, New York, United States of America; 5 Harvard-Partners Center for Genetics and Genomics, Boston, Massachusetts, United States of America; University of California, United States of America

## Abstract

The production of a functional mRNA is regulated at every step of transcription. An area not well-understood is the transition of RNA polymerase II from elongation to termination. The *S. cerevisiae* SR-like protein Npl3 functions to negatively regulate transcription termination by antagonizing the binding of polyA/termination proteins to the mRNA. In this study, Npl3 is shown to interact with the CTD and have a direct stimulatory effect on the elongation activity of the polymerase. The interaction is inhibited by phosphorylation of Npl3. In addition, Casein Kinase 2 was found to be required for the phosphorylation of Npl3 and affect its ability to compete against Rna15 (Cleavage Factor I) for binding to polyA signals. Our results suggest that phosphorylation of Npl3 promotes its dissociation from the mRNA/RNAP II, and contributes to the association of the polyA/termination factor Rna15. This work defines a novel role for Npl3 in elongation and its regulation by phosphorylation.

## Introduction

Gene expression is regulated through a complex series of events that coordinate the synthesis, processing and export, and in some instances, degradation of the mRNA. As the polymerase reaches the end of the gene, these events must coincide to produce a functional mRNA. The transcription of sequences that specify cleavage/polyadenylation signals directs the termination phase of transcription [1], [2]. In yeast, the cleavage/polyadenylation machinery, which is composed of the Cleavage Factor I (CFI) (CSTF in humans) and Cleavage/Polyadenylation Factor (CPF) (CPSF in humans) complexes, is responsible for the recognition of polyA/termination signals and their subsequent processing [2]–[6]. Within CFI, Rna15, the homolog to mammalian CSTF64, specifically recognizes and binds sequences that direct the polyA/termination machinery to the 3′ end [7]. Lacking is a detailed understanding of how the recognition of polyA signals transitions towards transcription termination, and how this is linked to the preceding elongation phase. The allosteric or anti-termination model takes into account some of these events. In this model, association of a positive elongation factor or an anti-terminator with RNA Polymerase II (RNAP II) is essential. Dissociation of this factor from the polymerase at the end of a gene is predicted to have two effects: (1) it destabilizes transcriptionally active RNAP II, and (2) it promotes the recruitment of the polyA/termination machinery.

The SR-like protein Npl3 functions in transcription, 3′ end processing, hnRNP formation, and mRNA export [8]–[12]. Previously, we proposed that the competition between Npl3 and Rna15 for binding to RNA targets is central to the function of Npl3 in termination and 3′ end processing [11], [12]. Increased binding of Npl3 to a target sequence can shift the competition away from processing, strongly inhibiting cleavage/polyadenylation *in vitro* [12]. *In vivo,* mutations or deletion of *npl3* can result in the utilization of weak or otherwise poorly recognized polyA sites, while strong polyA signals are recognized and stably bound by cleavage/polyadenylation factors for processing [11], [13]. As we demonstrated previously, shifting the competition in favor of protection instead of processing by overexpressing Npl3 strongly inhibits cleavage/polyadenylation [12]. These results are consistent with a model of competition driven by the relative binding affinities of Rna15 and Npl3, yet an additional layer of regulation could not be excluded.

Phosphorylation plays an important role in the association/dissociation of a number of activators and factors that regulate transcription and mRNA processing. Most notable is the co-transcriptional phosphorylation of the carboxy terminal domain (CTD) of RNAP II. In *S. cerevisiae,* phosphorylation of the CTD repeat at serine 5 recruits capping enzyme, while phosphorylation at serine 2 functions to coordinate transcription and mRNA processing [14]–[17]. Another prominent transcription/RNA processing kinase is Casein Kinase 2 (CK2). In yeast, CK2 associates with elongation factors Spt16/Pob3 and Chd1 [18], [19]. In higher eukaryotes, one third of CK2 phosphorylation targets are involved in gene expression, half of these being transcription factors [20]. These include the activator PC4 (Sub1 in yeast), which is required for the regulation of mammalian promoter-dependent elements (DPE), and was shown to have an anti-termination effect [21]–[23]. CK2 also phosphorylates factors within the polyA/termination machinery that are important for 3′ end processing [24], [25].

Here we show that Npl3 directly interacts with phosphorylated serine 2 of the CTD and stimulates the elongation rate of RNAP II *in vitro.* Association with polymerase is inhibited by phosphorylation of Npl3 on S411. Stimulation of elongation is also reduced by a mutation in the Npl3 RNA binding domain, indicating that RNA binding is important. CK2 is shown to be required for Npl3's phosphorylation, and alter the competition between Npl3 and Rna15 for binding to the RNA. These results demonstrate that Npl3 functions as both a positive transcription elongation factor and an anti-terminator and that these activities are regulated by phosphorylation.

## Results

### Npl3 affects the rate of transcription elongation

**Figure 1:**
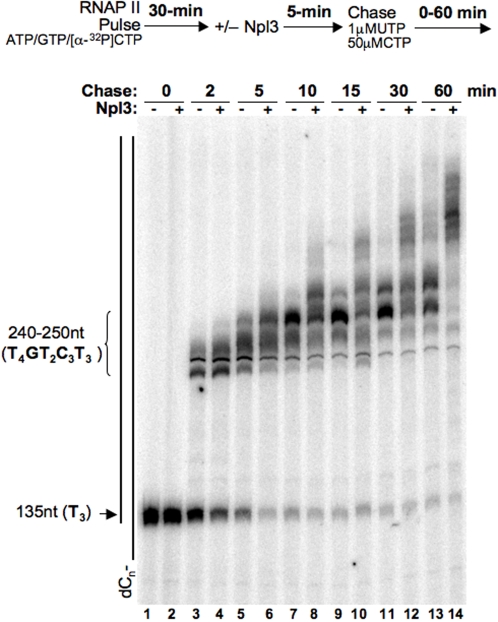
Npl3 stimulates RNAP II elongation. Oligo(dC)-tail templates were used in transcription reactions with or without 78 nM Npl3, as indicated. Reactions were pulsed by the addition of ATP, GTP, and *α* ^32^P-labeled CTP for 30 minutes as described in Materials & Methods. Transcripts were chased by the addition of excess CTP and limiting UTP for the indicated times. A schematic of the experimental scheme is shown on top. The oligo(dC)-template is represented next to the gel and the positions of the pause sites at stretches of Ts are indicated.

Mutations in or near the second RNA Recognition Motif (RRM) of *NPL3* have been shown to enhance termination [11]. These same *npl3* alleles confer sensitivity to MPA (mycophenolic acid) and 6AU (6-azauracil), phenotypes suggestive of elongation defects. To test for an effect of Npl3 on the rate of RNAP II elongation, an *in vitro* transcription assay containing purified RNAP II and a promoterless dC-tailed template was used [26]–[28]. In this assay, the polymerase binds to the single-stranded oligo(dC)-tail and initiates transcription in the presence of ATP/GTP/[*α*-^32^P]CTP [26]. In the absence of UTP, the polymerase is forced to pause at the first non-template T stretch (135 nt = T_3_) **(** **Figure 1, lanes 1 and 2** **)**. After a 30-minute labeling incubation, the reaction is split and incubated for an additional 5-minutes with buffer or Npl3. The labeled transcripts are then extended with limiting UTP and excess unlabeled CTP for 2 to 60 minutes in the absence **(** **Figure 1, odd lanes** **)**, or presence of Npl3 **(** **Figure 1, even lanes** **)**. Limiting the chase lowers the rate of nucleotide addition through dTTP regions on the template, causing the accumulation of transcripts that are ∼250 nt in length. These products are eventually chased into longer products. A notable increase in the rate of elongation by RNAP II was observed in the presence of Npl3 **(** **Figure 1** **)**. These results suggest that Npl3 functions as a positive elongation factor in the absence of termination factors.

**Figure 2:**
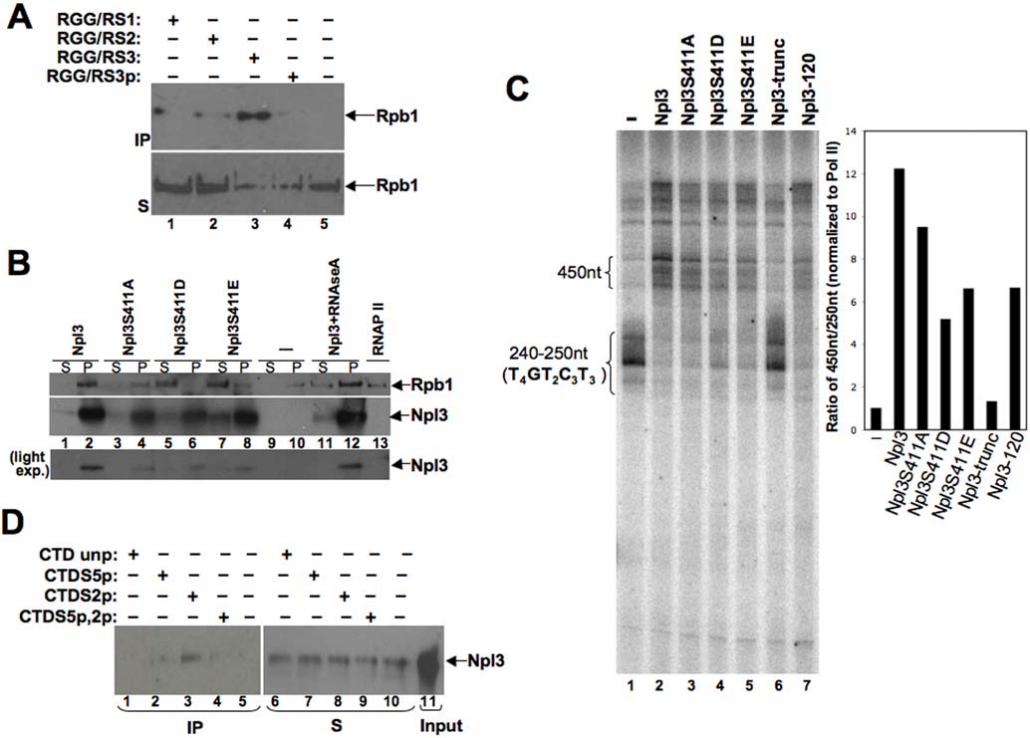
Npl3 interaction with RNAP II is affected by S411 phosphorylation. (A) Unphosphorylated peptide RGG/RS1 (lane 1), RGG/RS2 (lane 2), RGG/RS3 (lane 3) and phosphorylated RGG/RS3 (lane 4) were used in pull-down assays with purified RNAP II, as indicated, and one fifth of the input is shown in lane 5. The precipitated sample (P) and supernatant (S) were analyzed by immunoblotting. (B) Full-length Npl3 or S411 mutants were used in immunoprecipitation assays with purified RNAP II (100 ng), as indicated. A control lane with RNAP II (10 ng) is shown in lane 13. (C) Oligo(dC)-tail transcription reactions were performed as described in Figure 1 for 30 minutes with RNAP II only (lane 1) or with equivalent concentrations of Npl3 (lane 2); Npl3-S411A (lane 3); -S411D (lane 4); -S411E (lane 5); Npl3-truncated (RRMs only, aa 121–280) (lane 6); or Npl3-120 (lane 7). Quantification of the transcription reactions was performed by calculating the ratio of the 450 nt to the 250 nt bands and normalized using the RNAP II only lane, as shown in the graph. The experiment was done twice, and a representative gel was chosen. (D) Unphosphorylated (lane 1 and 6), or phosphorylated Ser 5 (lane 2 and 7), Ser 2 (lane 3 and 8), and Ser 2/Ser 5 (lane 4 and 9) CTD peptides were used in pull-down assays with full-length Npl3, as indicated, and input is shown in lane 11. The IP and S shown were analyzed as described for (C) using antibodies specific for Npl3. Control Npl3 with no peptide is shown in lanes 5 and 10.

### Unphosphorylated Npl3 interacts with RNAP II and mediates the elongation effect

The C-terminal RS domain of Npl3 contains three serine/proline [29] and six serine/arginine (SR) repeats that are interspersed within a region rich in arginines and glycines (RGG/RS domain). RS domains in other proteins can mediate protein-protein interactions [30]. Phosphorylation of RS domains also functions in the recruitment of these proteins to sites of transcription (*e.g.,* SF2/ASF) [29]. In addition, RS domain phosphorylation can affect RNA binding affinity [8], [30], [31].

Npl3 and RNAP II co-immunoprecipitate from yeast extracts, although it is unclear if this interaction is direct or indirect [9]. In order to test the role of the RGG/RS domain of Npl3 plus its phosphorylation in this interaction, peptides corresponding to three repeats within the RGG/RS domain were conjugated to a resin and used in pull-down experiments with purified RNAP II **(** **Figure 2A** **)**. Peptide RGG/RS1 corresponds to the SR repeat (GGYGGYSRGGYGGY), peptide RGG/RS2 corresponds to the SP repeat (RGGYDSPRGGY), and peptide RGG/RS3 corresponds to the SP repeat containing serine 411 (YRTRDAPRERSPTR). Since S411 is phosphorylated *in vivo,* and it has been extensively characterized [10], [32], we also included a phosphorylated form of this peptide (YRTRDAPRERpSPTR). No RNAP II was detected as interacting with the RGG/RS1 or RGG/RS2 peptides **(** **Figure 2A** **)**. However, significant binding was observed with the unphosphorylated RGG/RS3 peptide. Binding to the phosphorylated RGG/RS3 was much weaker. These results suggest that Npl3 interacts directly with RNAP II primarily through RGG/RS3 in its unphosphorylated form.

The contribution of S411 in RGG/RS3 to the interaction between Npl3 and RNAP II was further tested by immunoprecipitation with full-length Npl3 and three different mutants of S411 (S411A, S411E and S411D), which resemble unphosphorylated or phosphorylated forms of the protein. To rule out possible conformational changes due to the mutations, the point mutants were tested for RNA binding and found to retain their ability to bind RNA (data not shown). Purified RNAP II was incubated with recombinant histidine-tagged Npl3, Npl3-S411A, -S411D or -S411E, and reactions were immunoprecipitated using anti-His antibody and immunoblotted for RNAP II. Wild-type Npl3 bound to RNAP II **(** **Figure 2B** **)**, while mutants Npl3-S411A, -S411D and -S411E showed further decrease. This result suggests that changes to S411 affect the interaction, particularly those mutations that resemble a phosphorylated state.

The interaction between unphosphorylated RGG/RS3-S411 and RNAP II suggested that Npl3's effect on elongation might be regulated through phosphorylation of S411. The three mutants of S411 (S411A, S411E and S411D) were used in the elongation assay to test for this possibility. In addition, a mutant that was previously shown to have a defect in RNA binding (Npl3-120) and a truncated form of Npl3 containing only the two RRMs (aa 121–280) were also tested [11], [12], [33]. Elongation stimulation by Npl3-S411D and Npl3-S411E, which may resemble the phosphorylated form of Npl3, was reduced as compared to the wild-type **(** **Figure 2C** **)**. In addition, the Npl3-S411A mutant was slightly reduced. This result further highlights the importance of maintaining the integrity of the S411 residue since the S411A substitution differs only in the hydroxyl group present in the serine residue. These results are consistent with the model that S411 phosphorylation can modulate the effect on elongation. Notably, the truncated form of the protein did not stimulate elongation, which reinforces the importance of the RGG/RS domain and S411 in the interaction with RNAP II. Finally, the RRM mutant Npl3-120 also has reduced elongation stimulation, suggesting that RNA binding contributes to this activity. We conclude from these experiments that the positive effect of Npl3 on elongation requires both binding to the nascent RNA and its physical interaction with RNAP II, which may be inhibited by phosphorylation of S411.

### Npl3 interacts with phosphorylated serine 2 of the CTD

The phosphorylated form of serine 2 of the CTD repeat YSPTSPS is associated with elongation [17]. Previous crosslinking experiments demonstrated the presence of Npl3 throughout elongation [9], suggesting a possible link to the CTD and possibly phosphorylated serine 2. An interaction with the CTD was first tested using the RGG/RS peptides. As with purified RNAP II, the unphosphorylated RGG/RS3 was observed to interact with GST-CTD (data not shown). In addition, unphosphorylated or phosphorylated Ser 2, Ser 5 or Ser 2/Ser 5 CTD peptides were used in pull-down experiments with unphosphorylated full-length Npl3 **(** **Figure 2D** **)**. The CTD repeat peptide containing the Ser 2 phosphorylation was observed to interact with Npl3, and this result was reproducible. We concluded from these experiments that the phosphorylated form of Ser 2 CTD mediates the physical interaction between unphosphorylated Npl3 and RNAP II.

### Cka1 phosphorylates Npl3 *in vitro*

In yeast, the cytosolic kinase Sky1 phosphorylates the SP motif closest to the C-terminus of Npl3 (S411) [10]. Phosphorylation of Npl3 at S411 in a *sky1* deletion strain is reduced, but not abolished [8] (data not shown), raising the possibility that another Npl3-specific kinase remained to be identified, and that other phosphorylation sites in Npl3 might exist. To ascertain the later possibility, endogenous Npl3 was immunoprecipitated from whole cell extracts and phosphorylation sites were analyzed by mass spectrometry. Multiple phosphorylation sites were identified in the endogenous Npl3 **(** **Table 1** **)**. Our results confirmed phosphorylation of serine 224, 349 and 356, which where identified previously in two large-scale phosphorylation analyses [34], [35]. One new additional site was shown to be phosphorylated, serine 212. This data is summarized in **Table 1**.

**Table 1:** Npl3 phosphorylation sites.

Npl3 (414 aa)	Phosphorylated Serines	Treatment	Analysis	Reference
**Endogenous**	**Ser212,**	WCE (whole cell extract)	MS	This study
	Ser224	WCE	MS-IMAC	This study, [34]
	**Ser349, Ser356**	WCE	MS-IMAC	(Ficarro *et al*, 2002)
	Ser411	^32^P-*in vivo* labeling	2D gel	(Gilbert *et al*, 2001)
**CK2 in vitro phosphorylated**	Ser77, Ser79,	Kinase assay	MS	This study
	**Ser212**, Ser230,		“	
	Ser328, Ser336,		“	
	**Ser349, Ser356**		“	
	Ser411		Point mutants	

Phosphorylation sites identified in Npl3.

* Highlighted residues where phosphorylation sites detected in both the endogenous and *in vitro* CK2 phosphorylated Npl3.

**Figure 3:**
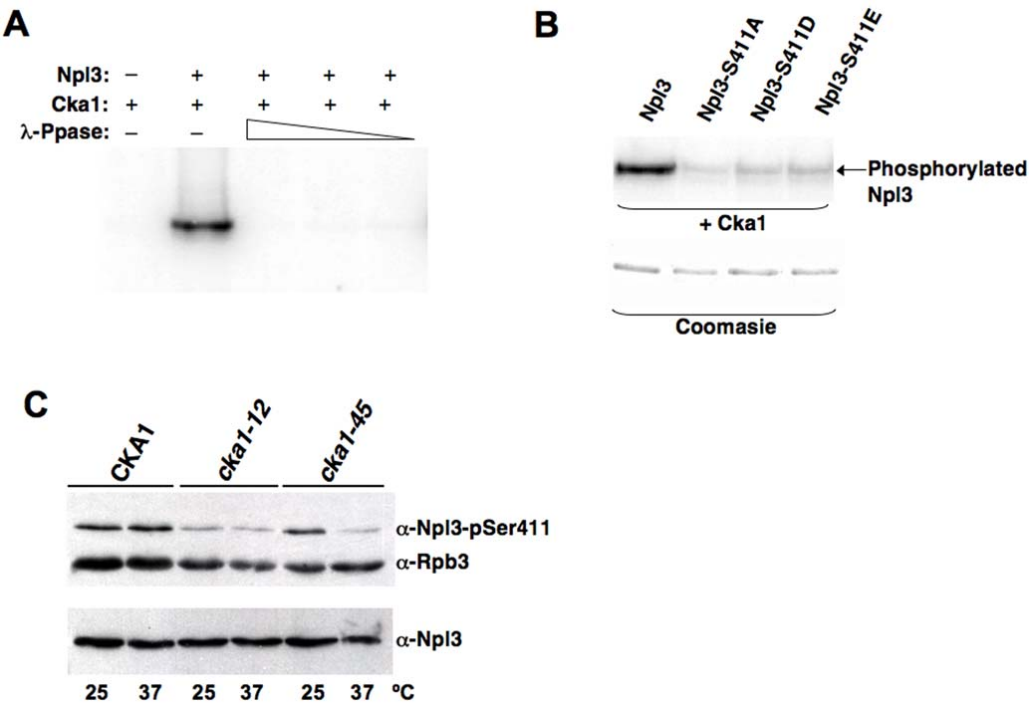
Cka1, the alpha catalytic subunit of CK2, phosphorylates Npl3. (A) Recombinant His-Cka1 was incubated with His-Npl3 in the presence of radiolabeled ATP. Phosphorylation of Npl3 is reversed by the addition of increasing concentrations of *λ*-Phosphatase. (B) Using the *in vitro* kinase assay with mutants of Npl3-S411, this residue was uncovered as an additional phosphorylation site. Wild-type His-Npl3 or S411 point mutants, His-Npl3-S411A, -S411D or -S411E were incubated with His-Cka1, as described for (A) in the presence of radiolabeled-ATP. Coomasie stain representing the concentration of recombinant Npl3 proteins used is shown below. (C) *CKA1* mutations show reduced phosphorylation of Npl3. Whole-cell extracts were prepared for cells grown for one hour at the permissive (25°C) or non-permissive (37°C) temperature for wild-type *CKA1, cka1-12* or *cka1-45*, and immunoblot analysis was performed using antibodies specific for phosphorylated or non-phosphorylated Npl3, as indicated. Detection of Rpb3 with specific antibodies is shown as a loading control.

We speculated that a putative kinase might be able to modulate the activity of Npl3 in the nucleus. As noted earlier, CK2 kinase had been shown to phosphorylate several targets within the cleavage/polyadenylation machinery (Cft1, Fip1, Brr5 and Pta1) [24], [36]. Therefore, this kinase was a good candidate and was tested for its ability to phosphorylate Npl3 *in vitro*. Recombinant His-Cka1 and His-Npl3 were purified from *E. coli* and incubated in the presence of [*γ*-^32^P] labeled ATP. Cka1 phosphorylates Npl3 and this modification is reversed by addition of Lambda-Phosphatase **(** **Figure 3A** **)**. A combination of mass spectrometry and point mutants was used to further analyze phosphorylated peptides in Npl3. *In vitro*, CK2 phosphorylation resulted in modification of multiples sites in Npl3, including Ser 212, Ser 349 and Ser 356 sites identified *in vivo* **(** **Table 1** **)**. Phosphorylation of S411 was tested using the S411 substitutions (S411A, S411D and S411E) in the kinase assay **(** **Figure 3B** **)**. A significant decrease was observed in the phosphorylation of Npl3-S411A, -S411D and -S411E as compared to wild-type. The residual phosphorylation signal observed for the mutant Npl3s is probably due to phosphorylation of the other sites reported in **Table 1**. We conclude from these results that Npl3 is hyperphosphorylated *in vivo* and that S411 is likely a major target of CK2 phosphorylation.

Given that the phosphorylation of sites in Npl3 did not strictly fit the known consensus sequence for mammalian CK2 ([ST]XX[EDpTpS]) [20], and that the RGG/RS1-3 peptides were not efficiently phosphorylated when tested in the *in vitro* CK2 kinase assay (data not shown), the specificity of the yeast Cka1 was assayed by mass spectrometry using peptide libraries **(** **Figure S1** **)**. Quantification by mass spectrometry of the phosphorylation efficiency of Cka1 confirmed the specificity of yeast Cka1 used in our study for peptides with serine or threonine residues within acidic motifs, as previously reported for the mammalian CK2 counterpart [20], [37]. Conversely, peptides containing SP motifs were phosphorylated very poorly **(** **Figure S1** **)**. In the context of the entire Npl3 protein, the SP or SR motifs of Npl3 that were detected as phosphorylated *in vitro* might reside in an acidic environment that favors phosphorylation by Cka1. This possibility is supported by evidence from the structure of the central RRM domain of Npl3, which revealed that all acidic residues lie on the surface of the protein [33]. Npl3's reported self-association may also be a factor, especially given recent evidence that dimerization is in some instances required for the phosphorylation of targets with poor similarity to recognition motifs [38], [39].

### CK2 phosphorylates Npl3 *in vivo*

The ability of CK2 to phosphorylate Npl3 was next tested in the context of the cellular environment using strains that have the genes for both of the catalytic subunits of CK2 deleted and carry complementing plasmids for either a wild-type (*CKA1)* or a mutant allele of *CKA1* (*cka1-12*(*ts*) or *cka1-45*(*ts*)) [40]. These strains were grown and assayed for the steady state phosphorylation of Npl3 by immunoblotting with general anti-Npl3 and anti-phospho-S411, which was demonstrated previously to be specific for the S411 phosphorylation *in vivo* [10]. The wild-type *CKA1* strain showed robust phosphorylation of Npl3 at both permissive (25°C) and non-permissive temperatures (37°C), whereas *cka1-12* showed reduced phosphorylation at both temperatures, and *cka1-45* showed a decrease upon the shift to the non-permissive temperature **(** **Figure 3C** **)**. At steady-state-levels, Npl3 localizes to the nucleus [41]. Therefore, the phosphorylation decrease observed in the *cka1* mutant strains was assumed to be due to CK2 mutations. The residual phosphorylation of S411 is presumed to be due to other kinases, including Sky1. While indirect phosphorylation of Npl3 cannot be absolutely excluded, we conclude from these results that Cka1 is required for the phosphorylation of Npl3-S411 *in vivo*.

### Phosphorylation of Npl3 affects its competition for RNA binding

**Figure 4:**
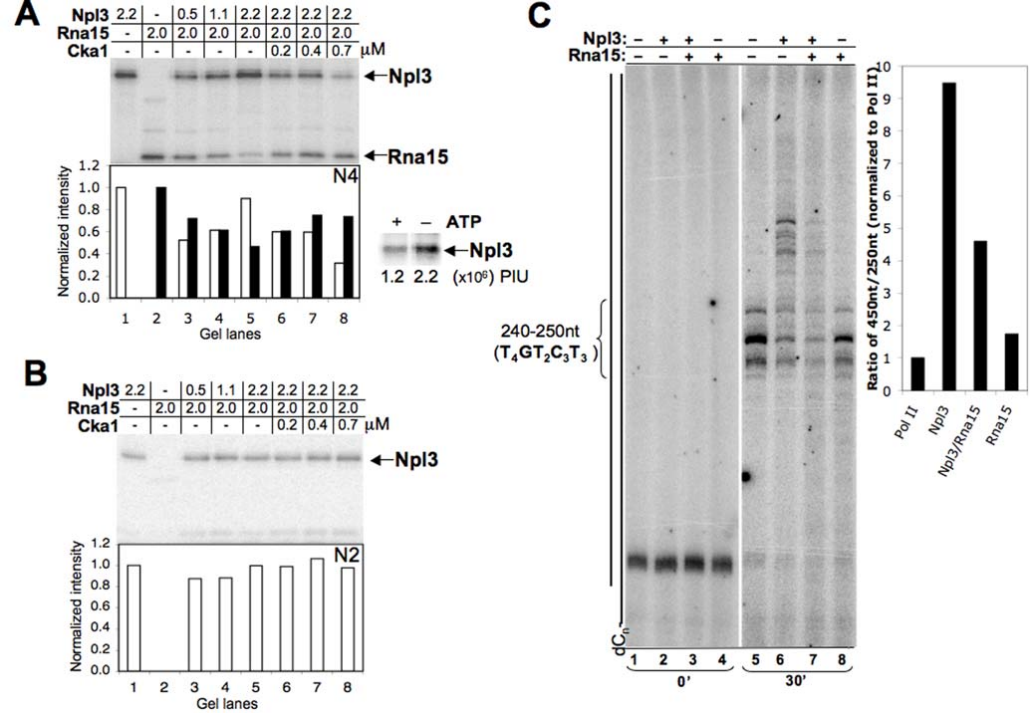
Cka1 disrupts the ability of Npl3 to effectively compete for binding to an Rna15-preferred sequence. (A) Recombinant Npl3, Rna15 and Cka1 were incubated with a radiolabeled RNA oligo (N4), UV cross-linked, and resolved in denaturing 10% SDS-PAGE gels. This RNA oligo consists of an A-rich repeat (described in Materials and Methods), which is the preferred binding site for Rna15 and is commonly found upstream of polyA sites [7]. Representative UV-cross-linking experiments are shown where increasing Cka1 is added to reactions containing Npl3 with Rna15. The graph below each gel shows quantification for the average of three experiments (Npl3, white bars; Rna15, black bars). Binding levels were calculated as fractions relative to a reaction containing the highest concentration of the individual RNA-bound protein (lane 1 for Npl3 and lane 2 for Rna15). Control UV-crosslinking experiment where Cka1 has been added with or without ATP is also shown. Values represent total PhosphorImager units (PIU). (B) Recombinant Npl3, Rna15 and Cka1 were incubated with a radiolabeled U/G/C-rich RNA oligo (N2), UV-cross-linked, and resolved in denaturing 10% SDS-PAGE gels and quantified as described for (A). (C) Similar reactions were performed as described for Figure 1 for 0 and 30 minutes without (lane 1 and 5) or with 78 nM Npl3 (lanes 2–3 for 0 min time-point; and 6–7 for the 30 min time-point). 200 nM Rna15 was added to the transcription reactions in the presence or absence of Npl3 (lanes 3–4 for 0 min time-point; and 7–8 for the 30 min time-point). The oligo(dC)-template is represented next to the gel and the positions of the pause sites at stretches of Ts are indicated. Quantification of the transcription reactions was performed as shown for Figure 2C.

As mentioned earlier, phosphorylation of Npl3 by Sky1 reduces RNA binding affinity [8]. Therefore, Npl3 phosphorylation might be expected to reduce its ability to compete with Rna15. Using a modified UV cross-linking assay with a labeled RNA oligo (N4) thought to be a preferred binding site for Rna15 at *GAL7* [7], recombinant Npl3 and Rna15 were tested for binding. Npl3 was observed to compete strongly for binding to the polyA signal **(** **Figure 4A** **)**. Titration of Cka1 resulted in an ATP-dependent reduction of Npl3 binding to the RNA, while binding to an oligo corresponding to an Npl3-preferred site was unaffected (N2) (**Figure 4B** **)**. No cross-linking of Rna15 was detected with the N2 oligo. Rna15 was not phosphorylated by Cka1, nor was binding to the polyA signal affected in reactions with Cka1 lacking Npl3 (data not shown). Thus, we concluded that phosphorylation of Npl3 by Cka1 had a dramatic effect on the RNA binding competition at Rna15-preferred sequences.

### Competition for RNA binding decreases stimulation of elongation

Previous characterization of alleles of *NPL3* demonstrated that the function of Npl3 in transcription termination relies on its ability to bind RNA [11], [33]. This defect is due to mutations in RRM2, which result in reduced the binding specificity of Npl3 [33], [42], and reduce the ability of the protein to compete effectively for binding to polyA/termination sequences [12]. In the elongation assay, the RRM mutant Npl3-120 had slightly reduced elongation stimulation, which suggested that RNA binding was also important for elongation **(** **Figure 2C** **)**. We exploited the unspecific RNA binding of Rna15 and its competition with Npl3 to test the RNA binding requirement of Npl3, presuming that binding of Rna15 to the nascent RNA might prevent a stable Npl3/RNA interaction. Stimulation of elongation was therefore measured in the presence of the competing polyA factor Rna15. A two-fold molar excess of Rna15 (relative to Npl3) was added together with Npl3 during the 5-min incubation between the pulse and chase. **Figure 4C** shows the results for transcription reactions for 0 and 30-minutes after UTP addition. Rna15 alone did not affect the rate of elongation (**Figure 4C, lane 8**). Npl3 still stimulates elongation in the presence of Rna15, but a decrease in the intensity of the signal for the longer transcripts was seen. Therefore, competition for binding to the RNA transcript between Rna15 and Npl3 may decrease the positive effect on elongation, and strengthens the suggestion that RNA binding plays an important role in the stimulatory effect of Npl3.

### Termination defects associated with Npl3 RNA binding and phosphorylation

Our previous chromatin immunoprecipitation experiments demonstrated that mutations in the RNA binding domain of Npl3, such as the *npl3-120* allele, lead to more efficient termination and increased cross-linking of polyA/termination factors to the 3′ end of various genes [11]. Phosphorylation of Npl3 reduces its association with RNA *in vivo* [10], and its association with an Rna15 preferred binding site *in vitro* **(** **Figure 4A** **and** [12]. Our results further suggest it is also important for regulating its interaction with RNAP II. Therefore, the expectation was that a mutation that abolishes S411 phosphorylation might result in a termination defect. The S411A mutation was selected for analysis since it more closely resembles an unphosphorylated state, although slight decreases in the elongation and binding assays were detected upon loss of the serine hydroxyl group. The predicted outcome *in vivo* was that a sustained interaction between unphosphorylated Npl3, the polymerase and RNA, would result in failure of RNAP II to release and terminate, and a more effective competition with Rna15 for recognition of termination/polyA signals, based on the assumption that both mechanisms are coupled *in vivo*. This prediction was tested by RNA expression analysis using whole genome tiled arrays consisting of 25-mer reverse probes with 5 base pair spacing. RNA was extracted from *NPL3, npl3-120* and *npl3-S411A* strains, followed by synthesis of labeled sense-strand cDNA and hybridization to the tiled arrays [43]. The intensities of two independent experiments were used to plot the ratio of mutant to wild-type.

**Figure 5:**
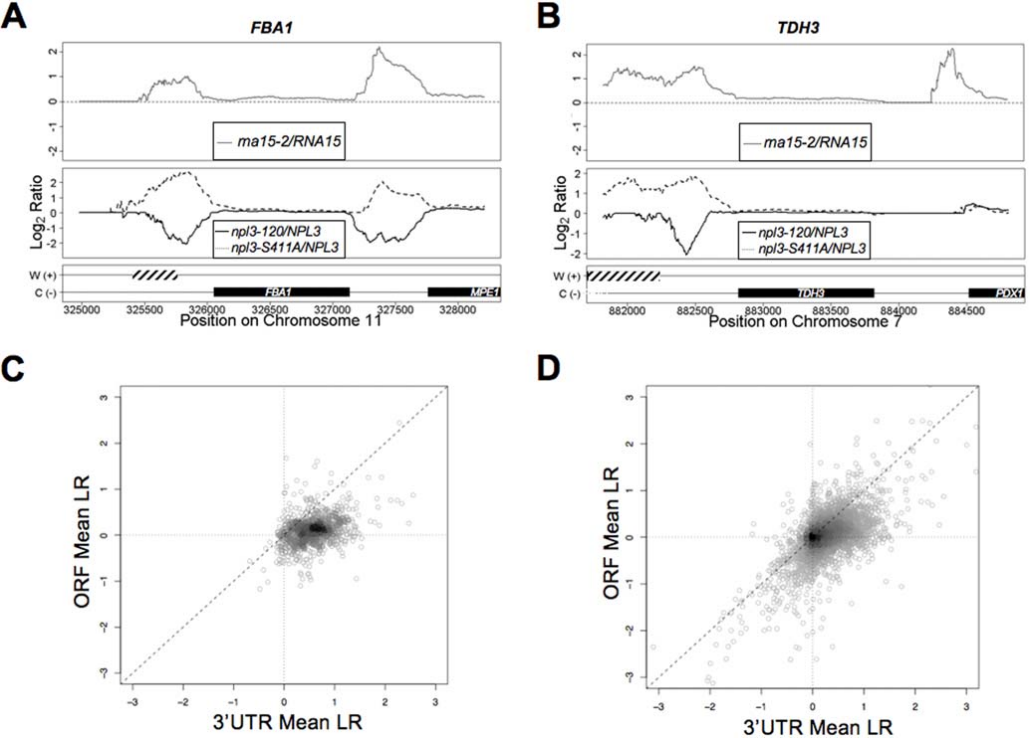
Opposing effects in termination demonstrated using RNA-binding or phosphorylation defective *npl3* alleles. RNA was extracted from *NPL3, npl3-120* and *npl3-S411A* and whole transcript sense strand cDNA was synthesized and used for hybridization to tiled arrays. The hybridization signals for (A) *FBA1*, *MPE1* and (B) *TDH3* for two independent experiments were used to calculate the ratio of mutant *vs.* wild-type. The top panel shows the ratio of *rna15-2/RNA15;* bottom panel shows the ratio of each *npl3* mutant*/NPL3*; a solid line for *npl3-120/NPL3*, or dashed line for *npl3-S411A/NPL3.* The corresponding position and orientation (Watson (W+), dashed bars, or Crick (C-) strand, black solid bars) of the genes is also shown. (C–D) A 300 bp region at the beginning of each ORF (+50 to +350 relative to the start codon), was selected for comparison to the same size region at the 3′UTR (−100 to +200 relative to the stop codon) of the genes that showed increase readthrough for the *npl3-S411A* strain. The mean value of the log_2_ ratio of *npl3-S411A/NPL3* was calculated and graphed in a scatter plot. (D) The total number of genes corresponding to the Crick strand were used to calculate the mean value of the same region described for (C).

Shown in **Figure 5A–B** are plots for three representative genes (*FBA1*, *MPE1* and *TDH3*). The ratio of mutant to wild-type expression levels showed a clear difference at the 3′ ends. For the *npl3-120* strain, a decrease in transcript levels near the 3′end of the genes was observed **(** **Figure 5** **and** **Figures S2** **,** **S3** **and** **S4** **)**, consistent with previous experiments showing that this mutation leads to more efficient termination [11]. In contrast, the *npl3-S411A* mutant had the opposite effect, showing increased hybridization at the 3′ end, indicating the presence of readthrough transcription beyond the polyA site. A list of genes with a similar transcription readthrough defect for the *npl3-S411A* strain and additional sample plots are included in **Table S2** **and** **Figures S2** **,** **S3** **and** **S4**. For reference, the plot corresponding to the ratio of the *rna15-2 vs.* wild-type is included. The *rna15-2* strain has a well-characterized termination defect that results in readthrough transcription [44]–[46]. At the *FBA1* gene, changes were observed in the *npl3-S411A* and *rna15-2* mutants at both ends of the gene denoting a terminating effect in the flanking *MPE1* gene **(** **Figure 5A** **)**. For two of the examples shown (*FBA1* and *MPE1*), the ratio of *rna15-2* strain was similar to that of the *npl3-S411A* **(** **Figure 5A** **)**. The *PDX1* gene lying upstream of *TDH3,* had a clear termination defect in the *rna15-2* strain, but was unaffected by the *S411A* or *npl3-120* mutations **(** **Figure 5B** **)**. This suggests that the competition between Npl3 and Rna15 might be dependent on *cis-*elements; *i.e.,* strong polyA signals may override the competition [12]. The *npl3-S411A* mutation resulted clearly in readthrough transcription of >800 genes **(** **Table S2** **)**. A scatter-plot is provided for these genes using the mean value for the log_2_ ratio of *npl3-S411A vs.* wild-type for a 300 bp region within the ORF and at the 3′UTR of each gene **(** **Figure 5C** **)**. An increase in mean value of the probes was observed at the 3′UTR. A scatter-plot in **Figure 5D** was also generated for the total number of genes in the Crick strand. The majority of these genes showed an increase mean value at the 3′UTR relative to the region within the ORF for the *npl3-S411A* mutant **(** **Figure 5D** **)**. Our results are consistent with the involvement of Npl3 in a widely distributed mechanism of transcription termination.

## Discussion

Termination of transcription is a critical mechanistic step in regulating gene expression, particularly for genes with multiple polyadenylation sites. Here we report that the SR/hnRNP protein Npl3 directly enhances the elongation rate of RNAP II. Although co-transcriptional recruitment of eukaryotic RNA binding proteins is now well established, to our knowledge a direct stimulation of elongation by SR or hnRNP proteins has not been previously reported. No such stimulation is seen with the RNA binding protein Rna15 or the truncated Npl3 RRM domain, arguing that the Npl3 effect is not due to non-specific interaction with RNA.

**Figure 6:**
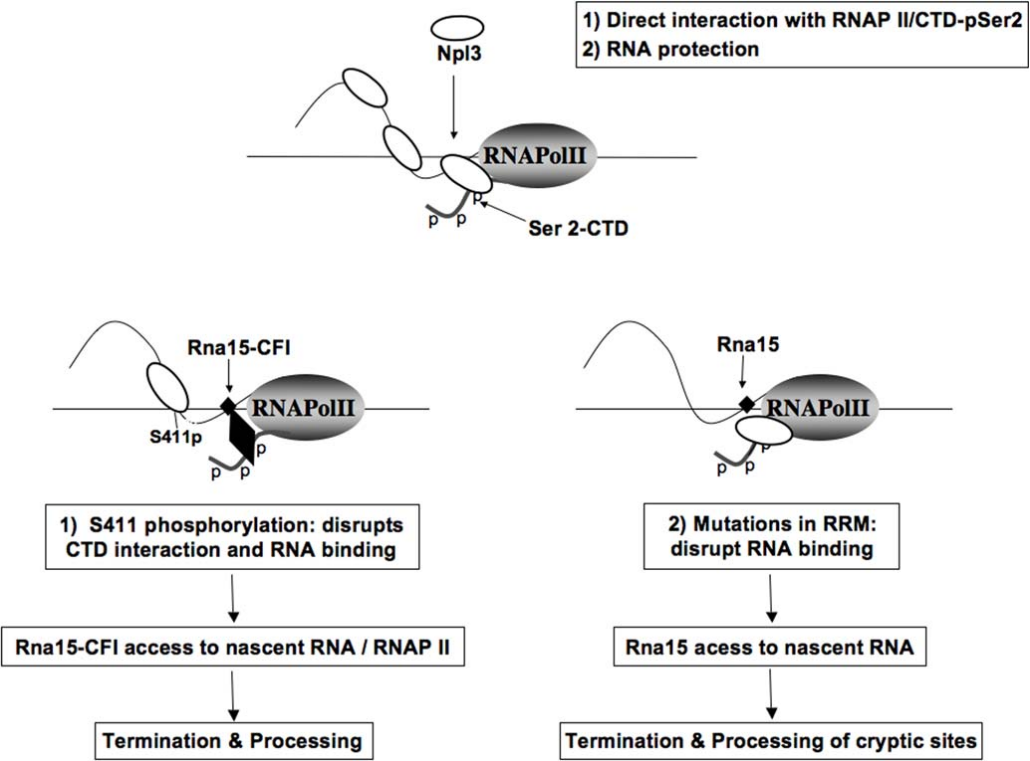
Two activities of Npl3 promote elongation. Npl3 directly stimulates the elongation rate of RNAP II by physically interacting with phospho-Ser 2 of the CTD and the RNA. Phosphorylation regulates Npl3's interaction with RNAP II and RNA, and promotes binding of polyadenylation/termination factors. Binding of Npl3 to the nascent RNA may stabilize interactions between CTD-RNAP II and the RNA. Npl3, white circle; Rna15, small black diamond; CFI, large black diamond; RNAPII, grey shaded oval.

The data presented is consistent with a model where Npl3 functions in two ways: it directly interacts with phosphorylated serine 2 of the polymerase CTD to promote elongation and at the same time binds RNA to antagonize binding of the polyA/termination factor Rna15 **(** **Figure 6** **)** [9], [11]. This combination of activities resembles the l-related phage 82 anti-terminator Q^82^ that, together with the NusA protein, decreases pausing of the bacterial RNA polymerase and protects the RNA transcript from termination factors [47].

Phosphorylation of Npl3 reduces its interaction with RNAP II and RNA. It is interesting to speculate that the targeted phosphorylation of Npl3 at 3′ ends of genes might help trigger termination. This model is supported by chromatin immunoprecipitation experiments where crosslinking of Npl3 was observed to decrease downstream of the polyA site [11], by the expression analysis presented here where mutations at S411 or in the second RRM of Npl3 were shown to specifically affect transcript signals at 3′ ends of genes, and by UV-crosslinking where the Cka1 phosphorylation decreased Npl3's competition for binding to polyA signals. In addition, a recent report by Lund *et al* suggests that phosphorylation is involved the autoregulation of the *NPL3* transcript [32]. The cytosolic kinase Sky1 phosphorylates Npl3 [8]. Thus far, no other kinase had been linked to the phosphorylation of Npl3. CK2 is the only kinase reported to phosphorylate proteins involved in mRNA 3′ end formation. Phosphorylation of 3′ end processing factors Pta1 and Fip1 by CK2 affects their ability to cleave and polyadenylate the mRNA [24]. Therefore, the phosphorylation of Npl3 by CK2 shown here would support a targeted phosphorylation at the 3′ end. The existence of nuclear and cytosolic kinases that phosphorylate an mRNA shuttling protein at an identical residue suggests that the same phosphorylation switch, which functions by modulating protein-protein and -RNA interactions can be activated in different compartments of the cell. It remains to be determined what other kinases operate at the 3′ end or whether additional transcription-associated kinases can control the phosphorylation state of Npl3 to regulate its activity at sites of transcription.

The model postulates several events that would affect assembly of the polyA/termination machinery at the 3′ end **(** **Figure 6** **)**. First, upon dissociation of Npl3, the elongation rate of RNAP II would decrease to enhance the window of opportunity for polyA factors to bind to signals in the nascent RNA. It is known that the rate of transcription elongation can affect alternative splicing decisions [48], [49], so it is reasonable to assume that a similar relationship can exist between elongation rate and polyadenylation. Interestingly, the elongation promoting activity of Npl3 is weaker in an RNA binding mutant and is inhibited by Rna15, which suggests that binding to the RNA transcript by Npl3 is important for stimulating RNAP II. Binding of Npl3 to the nascent transcript might stabilize interactions between the CTD, RNAP II and the RNA and prevent premature 3′ end formation. Accordingly, interactions between the CTD of the mammalian RNAP II with the RNA were shown to be critical to suppress premature termination [50]. Additionally, binding of Npl3 to the RNA may prevent the formation of arrest-inducing secondary structures, or suppress pausing at cryptic polyA sites.

A second event upon dissociation of Npl3 would be a shift in the balance of the competition between Npl3 and Rna14/15 for RNA-binding. Several lines of evidence have shown that this competition is important for proper polyadenylation. Transcription readthrough of weakened polyA signals can be suppressed by mutation/deletion of Npl3 or Cbp20/80, or by overexpression of the polyadenylation factors Rna14 or Hrp1 [11]–[13]. This competition between Npl3 and Rna15 can be reconstituted *in vitro* [12], and here we show that this competition can be shifted by the CK2 phosphorylation of Npl3.

There are many examples of SR proteins being regulated by phosphorylation. In the case of Srp40, phosphorylation of the RS domain is required for sequence-specific RNA binding [51]. For another RS protein, ASF/SF2, phosphorylation enhances contacts essential for splicing [8], [30], [52]–[55]. The identification by mass spectrometry of multiple phosphorylated serines *in vivo* and *in vitro* suggests that Npl3 is hyperphosphorylated at the RRM and RGG/RS domains. In the case of Npl3-S411, its phosphorylation decreases crosslinking of the protein to RNA [8](this study), and also functions in autoregulation of the *NPL3* transcript [32]. Our study suggests that CK2 is required for the phosphorylation of S411, and we also show that this residue appears to affect Npl3's interaction with RNAP II. Thus, S411 participates in the binding for both RNA and RNAP II.

While this manuscript was in preparation Lund *et al* reported of an effect of Npl3 in autoregulation of its own transcript [32]. In this study, phosphorylation of Npl3-S411 was observed to increase termination efficiency, thus modulating its own protein levels. Our expression analysis suggests that the termination of *NPL3* represents a unique case likely shared by a limited number of genes. Previous work by Steinmetz and co-workers suggested that the choice of termination sites at the *NPL3* locus involved a Sen1 anti-termination pathway [56]. In addition, the phosphorylation decrease observed for S411 in the *cka1* alleles reported in this study did not significantly affect the steady state levels of Npl3, as observed by Lund and co-workers. Therefore, we believe that the autoregulation of Npl3 is likely the result of an activity that is distinct from that mediated by CK2. At this time, the mechanism by which Npl3 functions in its autoregulation by allowing the selection of an alternative termination site remains unresolved.

Our study suggests that Npl3 affects elongation and termination in a high percentage of genes (∼30%) making this a widely used mechanism in yeast that is likely to function in higher eukaryotes. We predict that metazoan SR-proteins with known functions in hnRNP formation, splicing and mRNA 3′end processing might also be able to affect elongation in a manner similar to Npl3.

## Materials and Methods

### Plasmids and Strains

Plasmids pSBEThis7-*NPL3* and pSBEThis7-*npl3-120* were described previously [11]. For plasmids pSBEThis7-*npl3-S411A,* -*npl3-S411D*, and -*npl3-S411E,* the ORF of *NPL3* was amplified by PCR using the oligo: 5′-CATGCCATGGCTGAAGCTCAAGAAACTCACG-3′ and 5′- CGCGGATCCGCTTACCTGGTTGGTG**C**TCTTTCACG-3′ for the S411A; or 5′- CGCGGATCCGCTTACCTGGTTGGT**TC**TCTTTCACG-3′ for the S411D; and 5′-CGCGGATCCGCTTACCTGGTTGG**GTC**TCTTTCACG-3′ for the S411E substitution, and cloned into *BamH*I/*Nco*I site of pSBEThis7. Plasmid pET21b-*CKA1*-His6 was described previously [24]. DNA template pAdGR220 used for transcription assays was described previously [28]. Yeast strains used are shown in Table S1. The *npl3-S411A* strain was kindly provided by M. Lund (UCSF).

### Protein Purifications

*E. coli* strain Rosetta (DE3) or BL21(DE3) were transformed with pSBEThis7-*npl3-S411A, -S411D*, *-S411E*, pET21b-*RNA15* or -*CKA1-His6* and purified as described previously [11]. Wild-type RNAP II was purified from yeast as described previously [27].

### Pull-down and immunoprecipitation assays

Npl3 peptides were conjugated to resin using the Carboxylink Immobilization Kit (Pierce). Peptides containing the amino acid sequences: GGYGGYSRGGYGGY (RGG/RS1), RGGYDSPRGGY (RGG/RS2), YRTRDAPRERSPTR (RGG/RS3), or YRTRDAPRERpSPTR (RGG/RS3p) were synthesized (BCMP/HMS Biopolymers Lab). Peptides were normalized by OD 280nm. 100 ng of purified RNAP II (A. Ponticelli) was incubated with each of the conjugated peptides overnight at 4°C in IPP-150 buffer (10 mM Tris [pH 7.9], 150 mM NaCl, 1 mM MgOAc, 2 mM CaCl_2_, 0.1% NP-40, 1 mM DTT with protease inhibitors: 1 µg/ml of aprotinin, leupeptin, antipain and pepstatin-A), and washed using buffer IPP-150 plus 0.01% SDS (sodium dodecyl sulfate) [24]. Amino-terminal biotinylated CTD peptides were previously described [57]. For immunoprecipitations, IgM agarose (Sigma) was conjugated to a His antibody. 100 ng of purified RNAP II was incubated with recombinant His-Npl3, -S411A, -S411D or -S411E for 2 hours at 4°C in IPP-150 buffer and washed as described above. Alternatively, 5 µl of streptavidin-coated magnetic beads (Dynal) were used to bind 1 µg of a CTD peptide repeat (YSPTSPS) containing unphosphorylated, Ser 2, Ser 5 or Ser 2/Ser 5 phosphorylated peptides. 100 ng of recombinant Npl3 was incubated with each CTD peptide and 500 ng Bovine Serum Albumin (BSA), as described previously [57]. For immunoprecipitation of endogenous Npl3 for mass spectrometry analysis, Protein A (Sigma) was conjugated to polyclonal rabbit anti-Npl3 (kindly provided by Lithgow, La Trobe University, Australia) and incubated with 5 mg of whole cell extracts prepared as described previously [58]. All protein was eluted by boiling in the presence of SDS-PAGE loading buffer and resolved using 8% SDS-PAGE. Immunoblotting was done using standard methods using polyclonal mouse anti-CTD (8WG16).

### Oligo(dC)-tailed template transcription assays

Transcription reactions were performed as described previously [28]. Briefly, reactions were carried out at room temperature in the presence of 20 mM HEPES-KOH, pH 7.9, 20 mM Tris-HCl, pH 7.9, 8 mM MgOAc, 100 mM KOAc, 1 µM DTT, 0.5 mg/ml BSA, 3% (vol/vol) glycerol, 8 U of RNasin, 100 ng template DNA and 100 ng RNAP II. Pulse was carried out by the addition of nucleotides (50 µM ATP, 50 µM GTP, 2 µM CTP, and 10 µCi [α-^32^P] CTP (3000Ci/mmol), followed by incubation for 30 minutes. Chase was performed by the addition of 1 µM UTP and 50 µM unlabeled CTP [28]. Reactions were stopped by the addition of 10 mM Tris-HCl, pH 7.2, 0.5 mM EDTA and 0.3 M NaCl, 0.2% SDS and 20 µg proteinase K. The reaction mixture was extracted once with phenol-chloroform-isoamyl alcohol. Transcripts were resolved on an 8% polyacrylamide–7 M urea gel and then visualized using a PhosphorImager.

### Cka1 Phosphorylation and Phosphatase *in vitro* Assays

500 ng of recombinant Npl3, Npl3-S411A, -S411D, -S411E and Rna15 were phosphorylated in kinase buffer (20 mM HEPES-KOH, pH 7.0, 7.5 mM MgOAc, 100 mM KOAc, 2 mM DTT, 20% glycerol) at 30°C for 60 min with [*γ*-^32^P] –ATP (or cold ATP for MS analysis) and 500 ng recombinant Cka1-His6. For protein phosphatase assays, reactions were carried out in 1× Lambda Phosphatase (*λ*-PPase) reaction buffer [13] with 500 ng of His-Npl3 and titrated (200–600 U) Lambda Protein Phosphatase [13]. These were then incubated at 30°C for 60 min and stopped by the addition of SDS sample buffer. Proteins were subjected to sodium dodecyl sulfate-polyacrylamide gel electrophoresis (SDS-PAGE) and detected using a PhosphorImager.

### Mass spectrometry analysis

For identification of endogenous phosphorylated peptides in Npl3, the proteinwas immunoprecipitated from whole cell extracts. This *in vivo* phosphorylated Npl3 and the *in vitro* phosphorylated protein were excised from Coomassie-stained gels, digested with trypsin, and analyzed by LC-MS/MS in a linear ion trap (LTQ) mass spectrometer. MS/MS spectra were searched against the *S. cerevisiae* protein database.

### Protein analysis

For the preparation of yeast whole-cell extracts, 10 ml of cells were grown overnight, inoculated to an optical density (600 nm) of 1.5, allowed to grow at the permissive (25°C) or non-permissive (37°C) temperature for 2 hours, and pellet. Glass beads were used to disrupt cells in lysis buffer (20 mM HEPES [pH 7.6], 200 mM potassium acetate [KOAc], 10% glycerol, 1 mM EDTA) supplemented with protease inhibitors (1 mM phenylmethylsulfonyl fluoride and 1 µg/ml of aprotinin, leupeptin, antipain and pepstatin-A). Equal amounts of protein were then resolved using SDS-PAGE. Immunoblotting was performed using standard methods with polyclonal rabbit anti-phosphorylated Npl3 (kindly provided by C. Guthrie, UCSF) and anti-Npl3. Polyclonal rabbit anti-Rpb3 was from Neoclone, Horseradish peroxidase-conjugated polyclonal goat anti-rabbit was from SouthernBiotech, and goat anti-mouse HRP antibodies were from Jackson ImmunoResearch Laboratories.

### UV cross-linking assays

UV cross-linking experiments were performed as described previously [12]. RNA oligos containing the sequences: 5′-UAAUAAUGACUAUAUAUG-3′ (N4) or 5′-UUGCCUGGUUGCCUGGUU-3′ (N2) were synthesized (Invitrogen), and [*α*-^32^P]ATP 5′ end-labeled using T4 Polynucleotide Kinase (Invitrogen) as described previously [33]. RNA (∼100,000 cpm) was mixed with recombinant Npl3, Rna15, and Cka1 in kinase buffer (200 mM HEPES-KOH, pH 7.0, 75 mM MgOAc, 1 M KOAc, 20 mM DTT, 20% glycerol) with 200 µM ATP. All reactions were incubated for 20 minutes at room temperature and then UV irradiated in an Ultra-lum/UVC-515 ultraviolet multilinker set at 1800 µJ (×100). Loading buffer was added and reactions were resolved on a 10% SDS-polyacrylamide gel (30∶0.8 acrylamide∶bis), dried and exposed to a PhosphorImager screen.

### Expression analysis with tiled arrays

RNA for *NPL3, npl3-120, npl3-S411A* and *rna15-2* was extracted as described previously [59]. Sense RNA was synthesized using whole transcript sense target labeling assay (Affymetrix, HMS Biopolymers Facility), and hybridized to *S. cerevisiae* Tiling 1.0R Arrays, and intensities were analyzed using the Tiling Analysis Software (TAS) (https://www.affymetrix.com/support/learning/tiling_analysis/tiling_analysis_sw_tutorial.affx). Representative graphs were generated using the R software [60]. The microarray datasets in Figure 5 have been deposited in the GEO database (http://www.ncbi.nlm.nih.gov/projects/geo/) under accession number GSE12677.

## Supporting Information

Table S1 Yeast strains used in this study.
(0.04 MB DOC)

Table S2 Genes with increased Log2 Ratio at their 3′UTR.
(1.47 MB DOC)

Figure S1 Distribution of the log2 ratios of the peptide libraries phosphorylated in vitro with Cka1 and measured by MS. Kinase assays utilizing peptide libraries representing acidic (L[LE]D[KDN]D[DA][LE][ST]D[EL]E[LEN][EL]K) and proline-directed ([KP]L[VKE]L[AP][NE][ST]P[KI][LKP]VV[KL]) motifs were performed. 20 µg of the non-phosphorylated libraries were used, and 0.2 µg of the heavy phosphorylated standard library was added upon reaction quenching with 0.15 % TFA. Reactions were desalted in a tC18 SepPak and enriched for phosphopeptides with PhosSelect IMAC resin (Sigma). Samples were desalted again prior to injection into a capillary (125 µm×18 cm) C18 column and analyzed by LC-MS/MS in a LTQ-Orbitrap mass spectrometer using a 60 minute-gradient and data dependent TOP10 method. MS/MS spectra were searched against a database containing the sequences for all the peptides in the libraries and all the sequences from E. coli protein sequence database (used for distraction purposes) in the forward and reverse directions. Results were filtered to <1% false positives and peptides were further quantified using the VistaQUANT algorithm.
(0.07 MB TIF)

Figure S2 Opposing effects in termination demonstrated using RNA-binding or phosphorylation defective npl3 alleles. RNA was extracted from NPL3, npl3-120 and npl3-S411A and whole transcript sense strand cDNA was synthesized and used for hybridization to tiled arrays. Shown are the hybridization signals for twelve genes. The three top panels represent the individual expression intensities for each strain, with the black and gray dotted lines showing two independent experiments. The bottom panel shows the ratio of each mutant vs. wild-type; a solid line for npl3-120 vs. NPL3, or dashed line for npl3-S411A vs. NPL3, and the corresponding position and orientation (Watson (W+), shown for reference only, or Crick (C-) strand) of the genes (black solid bars).
(0.17 MB TIF)

Figure S3 Opposing effects in termination demonstrated using RNA-binding or phosphorylation defective npl3 alleles. RNA was extracted from NPL3, npl3-120 and npl3-S411A and whole transcript sense strand cDNA was synthesized and used for hybridization to tiled arrays. Shown are the hybridization signals for twelve genes. The three top panels represent the individual expression intensities for each strain, with the black and gray dotted lines showing two independent experiments. The bottom panel shows the ratio of each mutant vs. wild-type; a solid line for npl3-120 vs. NPL3, or dashed line for npl3-S411A vs. NPL3, and the corresponding position and orientation (Watson (W+), shown for reference only, or Crick (C-) strand) of the genes (black solid bars).
(0.18 MB TIF)

Figure S4 Opposing effects in termination demonstrated using RNA-binding or phosphorylation defective npl3 alleles. RNA was extracted from NPL3, npl3-120 and npl3-S411A and whole transcript sense strand cDNA was synthesized and used for hybridization to tiled arrays. Shown are the hybridization signals for twelve genes. The three top panels represent the individual expression intensities for each strain, with the black and gray dotted lines showing two independent experiments. The bottom panel shows the ratio of each mutant vs. wild-type; a solid line for npl3-120 vs. NPL3, or dashed line for npl3-S411A vs. NPL3, and the corresponding position and orientation (Watson (W+), shown for reference only, or Crick (C-) strand) of the genes (black solid bars).
(0.17 MB TIF)
