# Supplementary material for: Unphosphorylated SR-Like Protein Npl3 Stimulates RNA Polymerase II Elongation
Source: PLoS One. 2008 Sep 26;3(9):e3273. doi: 10.1371/journal.pone.0003273 (PMC2538588; doi:10.1371/journal.pone.0003273)
Supplement: Table S1 — Yeast strains used in this study. (0.04 MB DOC) [file pone.0003273.s001.doc]

# SUPPLEMENTARY TABLE 1

| Strain | Genotype |  |
| --- | --- | --- |
| MB11711 | Mat**α**, *his3-11, 15, his4-912δ*, *leu2Δ1, trp1-1* | This study |
| MB11712 | Mat**α**, *his4-912δ*, *lys2-128δ, leu2**Δ1, trp1-1, npl3-120* | This study |
| YSB451 | Mat**a,** *ura3-52*, *leu2Δ1*, *trp1Δ1*, *his3Δ200*, *cka1Δ1::HIS3*, *cka2Δ1::TRP1*, *ade2-101*, *lys2-801* [pRS316-*CKA1*] | (Sawa et al., 2004) |
| YSB465 | Mat**a**, *ura3-52*, *leu2Δ1*, *trp1Δ1*, *his3Δ200* *cka1Δ1::HIS3*, *cka2Δ1::TRP1*, *ade2-101*, *lys2-801* [pRS315-*cka1-12* (G57D)] | (Sawa et al., 2004) |
| YSB466 | Mat**a**, *ura3-52*, *leu2Δ1*, *trp1Δ1*, *his3Δ200* *cka1Δ1::HIS3*, *cka2Δ1::TRP1*, *ade2-101*, *lys2-801* [pRS315-*cka1-45* (G24E) | (Sawa et al., 2004) |

Sawa, C., Nedea, E., Krogan, N., Wada, T., Handa, H., Greenblatt, J., and Buratowski, S. (2004). Bromodomain factor 1 (Bdf1) is phosphorylated by protein kinase CK2. Molecular and cellular biolo*gy* 24, 4734-4742.
