# Supplementary material for: Unphosphorylated SR-Like Protein Npl3 Stimulates RNA Polymerase II Elongation
Source: PLoS One. 2008 Sep 26;3(9):e3273. doi: 10.1371/journal.pone.0003273 (PMC2538588; doi:10.1371/journal.pone.0003273)
Supplement: Table S2 — Genes with increased Log2 Ratio at their 3′UTR. (1.47 MB DOC) [file pone.0003273.s002.doc]

**Supplementary Table 2. Genes with increased Log2 Ratio at their 3'UTR.**

| SegmentID | GeneName | Allias | **Chr.** | **Start Coor.** | **stop coor.** | **strand** | **Qualifier** |
| --- | --- | --- | --- | --- | --- | --- | --- |
| YAL056C-A |  | YAL058C-A | 1 | 39047 | 38697 | C | Dubious |
| YAL054C | ACS1 | FUN44 | 1 | 45023 | 42882 | C | Verified |
| YAL049C |  |  | 1 | 52596 | 51856 | C | Uncharacterized |
| YAL046C |  |  | 1 | 57386 | 57030 | C | Uncharacterized |
| YAL042C-A |  | YAL043C-A | 1 | 61609 | 61232 | C | Dubious |
| YAL040C | CLN3 | WHI1|FUN10|DAF1 | 1 | 67521 | 65779 | C | Verified |
| YAL036C | RBG1 | FUN11 | 1 | 76153 | 75044 | C | Verified |
| YAL032C | PRP45 | FUN20 | 1 | 84475 | 83336 | C | Verified |
| YAL031C | GIP4 | FUN21 | 1 | 87032 | 84750 | C | Verified |
| YAL029C | MYO4 | SHE1|FUN22 | 1 | 92271 | 87856 | C | Verified |
| YAL023C | PMT2 | FUN25 | 1 | 108552 | 106273 | C | Verified |
| YAL022C | FUN26 |  | 1 | 110431 | 108878 | C | Verified |
| YAL010C | MDM10 | FUN37 | 1 | 135667 | 134186 | C | Verified |
| YAL007C | ERP2 |  | 1 | 138347 | 137700 | C | Verified |
| YAL001C | TFC3 | TSV115|FUN24 | 1 | 151168 | 147596 | C | Verified |
| YAR019C | CDC15 | LYT1 | 1 | 175133 | 172209 | C | Verified |
| YJL208C | NUC1 |  | 10 | 41183 | 40194 | C | Verified |
| YJL204C | RCY1 |  | 10 | 53151 | 50629 | C | Verified |
| YJL200C | ACO2 |  | 10 | 58813 | 56444 | C | Verified |
| YJL190C | RPS22A | RPS24 | 10 | 75301 | 74909 | C | Verified |
| YJL180C | ATP12 |  | 10 | 88558 | 87581 | C | Verified |
| YJL168C | SET2 | EZL1 | 10 | 104422 | 102221 | C | Verified |
| YJL158C | CIS3 | CCW5|PIR4|CCW11 | 10 | 122944 | 122261 | C | Verified |
| YJL153C | INO1 |  | 10 | 135930 | 134329 | C | Verified |
| YJL151C | SNA3 |  | 10 | 136770 | 136369 | C | Verified |
| YJL147C |  |  | 10 | 142564 | 141416 | C | Uncharacterized |
| YJL139C | YUR1 |  | 10 | 152996 | 151710 | C | Verified |
| YJL138C | TIF2 |  | 10 | 154688 | 153501 | C | Verified |
| YJL137C | GLG2 |  | 10 | 156124 | 154982 | C | Verified |
| YJL131C |  |  | 10 | 165345 | 164275 | C | Uncharacterized |
| YJL125C | GCD14 | TRM61 | 10 | 186677 | 185526 | C | Verified |
| YJL094C | KHA1 |  | 10 | 254437 | 251816 | C | Verified |
| YJL093C | TOK1 | YPK1|YORK|YKC1|DUK1 | 10 | 256807 | 254732 | C | Verified |
| YJL084C | ALY2 |  | 10 | 277997 | 274857 | C | Verified |
| YJL081C | ARP4 | ACT3 | 10 | 285266 | 283797 | C | Verified |
| YJL069C | UTP18 |  | 10 | 312701 | 310917 | C | Verified |
| YJL063C | MRPL8 | HRD238 | 10 | 316470 | 315754 | C | Verified |
| YJL058C | BIT61 |  | 10 | 327868 | 326237 | C | Verified |
| YJL044C | GYP6 |  | 10 | 359671 | 358295 | C | Verified |
| YJL023C | PET130 |  | 10 | 398393 | 397350 | C | Verified |
| YJL020C | BBC1 | YJL021C|MTI1 | 10 | 402405 | 398932 | C | Verified |
| YJL015C |  |  | 10 | 407511 | 407137 | C | Dubious |
| YJL012C | VTC4 | YJL012C-A|PHM3 | 10 | 413393 | 411228 | C | Verified |
| YJL008C | CCT8 |  | 10 | 421657 | 419951 | C | Verified |
| YJR009C | TDH2 | GLD2 | 10 | 454674 | 453676 | C | Verified |
| YJR019C | TES1 | PTE1 | 10 | 468274 | 467225 | C | Verified |
| YJR021C | REC107 | MER2 | 10 | 469572 | 468548 | C | Verified |
| YJR041C | URB2 | NPA2 | 10 | 513756 | 510232 | C | Verified |
| YJR043C | POL32 |  | 10 | 517506 | 516454 | C | Verified |
| YJR045C | SSC1 | mtHSP70|ENS1 | 10 | 521595 | 519631 | C | Verified |
| YJR058C | APS2 | YAP17 | 10 | 545168 | 544725 | C | Verified |
| YJR062C | NTA1 | DEA1 | 10 | 554842 | 553469 | C | Verified |
| YJR065C | ARP3 | ACT4 | 10 | 559151 | 557802 | C | Verified |
| YJR076C | CDC11 | PSL9 | 10 | 576594 | 575347 | C | Verified |
| YJR083C | ACF4 |  | 10 | 583530 | 582601 | C | Verified |
| YJR088C |  |  | 10 | 587366 | 586488 | C | Uncharacterized |
| YJR090C | GRR1 | SDC1|SSU2|COT2|CAT80 | 10 | 594320 | 590865 | C | Verified |
| YJR102C | VPS25 | VPT25 | 10 | 619758 | 619150 | C | Verified |
| YJR125C | ENT3 |  | 10 | 655957 | 654731 | C | Verified |
| YJR126C | VPS70 |  | 10 | 658683 | 656248 | C | Verified |
| YJR129C |  |  | 10 | 665013 | 663994 | C | Uncharacterized |
| YJR139C | HOM6 |  | 10 | 690518 | 689439 | C | Verified |
| YJR140C | HIR3 | HPC1 | 10 | 695690 | 690744 | C | Verified |
| YJR143C | PMT4 |  | 10 | 700608 | 698320 | C | Verified |
| YJR151C | DAN4 |  | 10 | 715734 | 712249 | C | Verified |
| YKL215C |  |  | 11 | 30688 | 26828 | C | Uncharacterized |
| YKL211C | TRP3 |  | 11 | 38154 | 36700 | C | Verified |
| YKL188C | PXA2 | PAT1 | 11 | 88791 | 86230 | C | Verified |
| YKL187C |  |  | 11 | 91541 | 89289 | C | Uncharacterized |
| YKL164C | PIR1 | CCW6 | 11 | 142824 | 141799 | C | Verified |
| YKL152C | GPM1 |  | 11 | 164390 | 163647 | C | Verified |
| YKL149C | DBR1 | PRP26 | 11 | 168834 | 167617 | C | Verified |
| YKL138C | MRPL31 |  | 11 | 185686 | 185291 | C | Verified |
| YKL119C | VPH2 | VMA12|CLS10 | 11 | 218861 | 218214 | C | Verified |
| YKL103C | LAP4 | API|YSC1|APE1 | 11 | 247326 | 245782 | C | Verified |
| YKL102C |  |  | 11 | 248011 | 247706 | C | Dubious |
| YKL076C | PSY1 |  | 11 | 292865 | 292482 | C | Dubious |
| YKL075C |  |  | 11 | 293952 | 292600 | C | Uncharacterized |
| YKL065C | YET1 |  | 11 | 316701 | 316081 | C | Verified |
| YKL063C |  |  | 11 | 321162 | 320659 | C | Uncharacterized |
| YKL060C | FBA1 | LOT1 | 11 | 327131 | 326052 | C | Verified |
| YKL059C | MPE1 |  | 11 | 329087 | 327762 | C | Verified |
| YKL057C | NUP120 | RAT2 | 11 | 333613 | 330500 | C | Verified |
| YKL056C | TMA19 | MMI1|RBF18 | 11 | 334559 | 334056 | C | Verified |
| YKL032C | IXR1 | ORD1 | 11 | 381501 | 379708 | C | Verified |
| YKL022C | CDC16 |  | 11 | 396891 | 394369 | C | Verified |
| YKL021C | MAK11 |  | 11 | 398393 | 396987 | C | Verified |
| YKL014C | URB1 | NPA1 | 11 | 416556 | 411262 | C | Verified |
| YKL008C | LAC1 | DGT1 | 11 | 428194 | 426938 | C | Verified |
| YKR004C | ECM9 | YKR004C-A | 11 | 447679 | 446442 | C | Verified |
| YKR006C | MRPL13 | YK105 | 11 | 450860 | 450066 | C | Verified |
| YKR014C | YPT52 |  | 11 | 465714 | 465010 | C | Verified |
| YKR017C |  |  | 11 | 472992 | 471337 | C | Uncharacterized |
| YKR018C |  |  | 11 | 475543 | 473366 | C | Uncharacterized |
| YKR019C | IRS4 |  | 11 | 477706 | 475859 | C | Verified |
| YKR026C | GCN3 | AAS2 | 11 | 489298 | 488381 | C | Verified |
| YKR036C | CAF4 |  | 11 | 510275 | 508344 | C | Verified |
| YKR043C |  |  | 11 | 521354 | 520539 | C | Uncharacterized |
| YKR045C |  |  | 11 | 523611 | 523060 | C | Uncharacterized |
| YKR068C | BET3 |  | 11 | 570551 | 569970 | C | Verified |
| YKR081C | RPF2 |  | 11 | 592182 | 591148 | C | Verified |
| YKR085C | MRPL20 |  | 11 | 599323 | 598736 | C | Verified |
| YKR087C | OMA1 |  | 11 | 603874 | 602837 | C | Verified |
| YLL051C | FRE6 |  | 12 | 39470 | 37332 | C | Verified |
| YLL050C | COF1 |  | 12 | 40413 | 39803 | C | Verified |
| YLL040C | VPS13 | VPT2|SOI1 | 12 | 63644 | 54210 | C | Verified |
| YLL034C | RIX7 |  | 12 | 73145 | 70632 | C | Verified |
| YLL032C |  |  | 12 | 76746 | 74269 | C | Verified |
| YLL030C |  |  | 12 | 80696 | 80355 | C | Dubious |
| YLL023C |  |  | 12 | 98835 | 97996 | C | Verified |
| YLL022C | HIF1 |  | 12 | 100200 | 99043 | C | Verified |
| YLL018C | DPS1 |  | 12 | 111574 | 109901 | C | Verified |
| YLR006C | SSK1 |  | 12 | 163892 | 161754 | C | Verified |
| YLR008C | PAM18 | TIM14 | 12 | 166082 | 165576 | C | Verified |
| YLR016C | PML1 |  | 12 | 177415 | 176801 | C | Verified |
| YLR026C | SED5 |  | 12 | 196473 | 195451 | C | Verified |
| YLR027C | AAT2 | ASP5 | 12 | 198086 | 196830 | C | Verified |
| YLR028C | ADE16 |  | 12 | 201316 | 199541 | C | Verified |
| YLR029C | RPL15A | RPL10A | 12 | 202591 | 201977 | C | Verified |
| YLR034C | SMF3 |  | 12 | 211934 | 210513 | C | Verified |
| YLR035C | MLH2 |  | 12 | 214457 | 212370 | C | Verified |
| YLR039C | RIC1 |  | 12 | 228597 | 225427 | C | Verified |
| YLR044C | PDC1 |  | 12 | 234082 | 232391 | C | Verified |
| YLR045C | STU2 |  | 12 | 237704 | 235038 | C | Verified |
| YLR047C | FRE8 |  | 12 | 241408 | 239348 | C | Verified |
| YLR050C |  |  | 12 | 246073 | 245588 | C | Uncharacterized |
| YLR065C |  |  | 12 | 266919 | 266374 | C | Uncharacterized |
| YLR073C |  |  | 12 | 281622 | 281020 | C | Uncharacterized |
| YLR074C | BUD20 |  | 12 | 282457 | 281957 | C | Verified |
| YLR085C | ARP6 |  | 12 | 301990 | 300674 | C | Verified |
| YLR089C | ALT1 |  | 12 | 320016 | 318238 | C | Uncharacterized |
| YLR093C | NYV1 | MAM2 | 12 | 327416 | 326514 | C | Verified |
| YLR110C | CCW12 |  | 12 | 370099 | 369698 | C | Verified |
| YLR118C |  | APT1 | 12 | 385409 | 384726 | C | Verified |
| YLR130C | ZRT2 |  | 12 | 404063 | 402795 | C | Verified |
| YLR144C | ACF2 | PCA1|ENG2 | 12 | 432017 | 429678 | C | Verified |
| YLR146C | SPE4 |  | 12 | 433726 | 432824 | C | Verified |
| YLR151C | PCD1 |  | 12 | 442738 | 441716 | C | Verified |
| YLR155C | ASP3-1 | ASP3 | 12 | 470406 | 469318 | C | Verified |
| YLR157C | ASP3-2 | ASP3 | 12 | 474058 | 472970 | C | Verified |
| YLR158C | ASP3-3 | ASP3 | 12 | 483638 | 482550 | C | Verified |
| YLR160C | ASP3-4 | ASP3 | 12 | 487290 | 486202 | C | Verified |
| YLR163C | MAS1 | MIF1 | 12 | 493256 | 491868 | C | Verified |
| YLR165C | PUS5 |  | 12 | 495260 | 494496 | C | Verified |
| YLR166C | SEC10 |  | 12 | 498046 | 495431 | C | Verified |
| YLR192C | HCR1 |  | 12 | 539591 | 538794 | C | Verified |
| YLR209C | PNP1 |  | 12 | 561734 | 560799 | C | Verified |
| YLR218C |  |  | 12 | 573920 | 573468 | C | Uncharacterized |
| YLR229C | CDC42 |  | 12 | 604789 | 604214 | C | Verified |
| YLR254C | NDL1 |  | 12 | 644975 | 644406 | C | Verified |
| YLR272C | YCS4 | LOC7 | 12 | 687204 | 683674 | C | Verified |
| YLR277C | YSH1 | BRR5 | 12 | 699497 | 697158 | C | Verified |
| YLR280C |  |  | 12 | 704835 | 704485 | C | Dubious |
| YLR281C |  |  | 12 | 704962 | 704495 | C | Uncharacterized |
| YLR286C | CTS1 |  | 12 | 710138 | 708450 | C | Verified |
| YLR287C |  |  | 12 | 712060 | 710993 | C | Uncharacterized |
| YLR287C-A | RPS30A |  | 12 | 713160 | 712539 | C | Verified |
| YLR288C | MEC3 | PIP3 | 12 | 714906 | 713482 | C | Verified |
| YLR291C | GCD7 |  | 12 | 719464 | 718319 | C | Verified |
| YLR292C | SEC72 | SIM2|SEC67 | 12 | 720372 | 719791 | C | Verified |
| YLR293C | GSP1 | CST17|CNR1 | 12 | 721432 | 720773 | C | Verified |
| YLR294C |  |  | 12 | 722032 | 721703 | C | Dubious |
| YLR295C | ATP14 |  | 12 | 722375 | 722001 | C | Verified |
| YLR337C | VRP1 | YLR337W|MDP2|END5 | 12 | 805106 | 802653 | C | Verified |
| YLR347C | KAP95 | RSL1 | 12 | 826412 | 823827 | C | Verified |
| YLR370C | ARC18 |  | 12 | 862253 | 861717 | C | Verified |
| YLR387C | REH1 |  | 12 | 897672 | 896374 | C | Verified |
| YLR389C | STE23 |  | 12 | 902660 | 899577 | C | Verified |
| YLR396C | VPS33 | VPT33|VPL25|VAM5|SLP1|  PEP14|MET27|CLS14 | 12 | 912310 | 910235 | C | Verified |
| YLR397C | AFG2 | DRG1 | 12 | 914892 | 912550 | C | Verified |
| YLR398C | SKI2 |  | 12 | 919019 | 915156 | C | Verified |
| YLR401C | DUS3 |  | 12 | 924448 | 922442 | C | Verified |
| YLR418C | CDC73 |  | 12 | 958092 | 956911 | C | Verified |
| YLR433C | CNA1 | CMP1 | 12 | 1006005 | 1004344 | C | Verified |
| YLR452C | SST2 |  | 12 | 1041364 | 1039268 | C | Verified |
| YML125C | PGA3 |  | 13 | 21700 | 20762 | C | Uncharacterized |
| YML115C | VAN1 | LDB13|VRG8|VRG7 | 13 | 41794 | 40187 | C | Verified |
| YML114C | TAF8 | TAF65 | 13 | 43575 | 42043 | C | Verified |
| YML110C | COQ5 | DBI56 | 13 | 50954 | 50031 | C | Verified |
| YML107C | PML39 |  | 13 | 56269 | 55265 | C | Verified |
| YML105C | SEC65 |  | 13 | 58687 | 57866 | C | Verified |
| YML104C | MDM1 |  | 13 | 62322 | 58939 | C | Verified |
| YML101C-A |  | YML102C-A | 13 | 69726 | 69409 | C | Dubious |
| YML097C | VPS9 | VPT9|VPL31 | 13 | 79690 | 78335 | C | Verified |
| YML076C | WAR1 |  | 13 | 115347 | 112513 | C | Verified |
| YML071C | COG8 | DOR1 | 13 | 131572 | 129749 | C | Verified |
| YML062C | MFT1 | MFT52 | 13 | 148683 | 147505 | C | Verified |
| YML049C | RSE1 |  | 13 | 178305 | 174220 | C | Verified |
| YML035C | AMD1 | AMD3 | 13 | 208860 | 206428 | C | Verified |
| YML025C | YML6 |  | 13 | 225365 | 224406 | C | Verified |
| YML018C |  |  | 13 | 235952 | 234771 | C | Uncharacterized |
| YML006C | GIS4 |  | 13 | 258416 | 256092 | C | Verified |
| YML004C | GLO1 |  | 13 | 262685 | 261705 | C | Verified |
| YMR008C | PLB1 |  | 13 | 282584 | 280590 | C | Verified |
| YMR013C | SEC59 |  | 13 | 296737 | 295178 | C | Verified |
| YMR015C | ERG5 | CYP61 | 13 | 302484 | 300868 | C | Verified |
| YMR016C | SOK2 |  | 13 | 305592 | 303235 | C | Verified |
| YMR021C | MAC1 | CUA1 | 13 | 318417 | 317164 | C | Verified |
| YMR029C | FAR8 |  | 13 | 330230 | 328659 | C | Verified |
| YMR036C | MIH1 |  | 13 | 343519 | 341855 | C | Verified |
| YMR039C | SUB1 | TSP1 | 13 | 349521 | 348643 | C | Verified |
| YMR049C | ERB1 |  | 13 | 370516 | 368093 | C | Verified |
| YMR088C | VBA1 |  | 13 | 445101 | 443413 | C | Verified |
| YMR089C | YTA12 | RCA1 | 13 | 448085 | 445608 | C | Verified |
| YMR099C |  |  | 13 | 464826 | 463933 | C | Verified |
| YMR110C | HFD1 |  | 13 | 491991 | 490393 | C | Verified |
| YMR111C |  |  | 13 | 493791 | 492403 | C | Uncharacterized |
| YMR114C |  |  | 13 | 497448 | 496342 | C | Verified |
| YMR121C | RPL15B |  | 13 | 510347 | 509733 | C | Verified |
| YMR127C | SAS2 |  | 13 | 523344 | 522328 | C | Verified |
| YMR131C | RRB1 |  | 13 | 534697 | 533162 | C | Verified |
| YMR132C | JLP2 |  | 13 | 535569 | 534943 | C | Uncharacterized |
| YMR146C | TIF34 |  | 13 | 558523 | 557480 | C | Verified |
| YMR164C | MSS11 |  | 13 | 589549 | 587273 | C | Verified |
| YMR165C | PAH1 | SMP2 | 13 | 592627 | 590039 | C | Verified |
| YMR166C |  |  | 13 | 594472 | 593366 | C | Uncharacterized |
| YMR168C | CEP3 | CSL1|CBF3B|CBF3 | 13 | 599157 | 597331 | C | Verified |
| YMR209C |  |  | 13 | 687283 | 685910 | C | Uncharacterized |
| YMR212C | EFR3 |  | 13 | 693042 | 690694 | C | Verified |
| YMR221C |  |  | 13 | 715444 | 713930 | C | Uncharacterized |
| YMR224C | MRE11 | NGS1|XRS4|RAD58 | 13 | 720652 | 718574 | C | Verified |
| YMR226C |  |  | 13 | 722395 | 721592 | C | Verified |
| YMR227C | TAF7 | TAF67 | 13 | 724384 | 722612 | C | Verified |
| YMR243C | ZRC1 | OSR1 | 13 | 756165 | 754837 | C | Verified |
| YMR244C-A |  |  | 13 | 758830 | 758516 | C | Uncharacterized |
| YMR247C | RKR1 |  | 13 | 768038 | 763350 | C | Verified |
| YMR258C |  |  | 13 | 784280 | 782619 | C | Uncharacterized |
| YMR259C |  |  | 13 | 788882 | 784620 | C | Uncharacterized |
| YMR265C |  |  | 13 | 797924 | 796539 | C | Uncharacterized |
| YMR268C | PRP24 |  | 13 | 804221 | 802887 | C | Verified |
| YMR274C | RCE1 |  | 13 | 815310 | 814363 | C | Verified |
| YMR275C | BUL1 | SMM2|RDS1|DAG1 | 13 | 818580 | 815650 | C | Verified |
| YMR290C | HAS1 |  | 13 | 851590 | 850073 | C | Verified |
| YMR293C |  |  | 13 | 856792 | 855398 | C | Uncharacterized |
| YMR301C | ATM1 |  | 13 | 869626 | 867554 | C | Verified |
| YMR310C |  |  | 13 | 896668 | 895715 | C | Uncharacterized |
| YMR311C | GLC8 |  | 13 | 897602 | 896913 | C | Verified |
| YNL331C | AAD14 |  | 14 | 17248 | 16118 | C | Verified |
| YNL316C | PHA2 |  | 14 | 43074 | 42070 | C | Verified |
| YNL305C |  |  | 14 | 59791 | 58898 | C | Uncharacterized |
| YNL302C | RPS19B | RP55B | 14 | 62943 | 61958 | C | Verified |
| YNL294C | RIM21 | PAL2 | 14 | 80260 | 78659 | C | Verified |
| YNL291C | MID1 |  | 14 | 85815 | 84169 | C | Verified |
| YNL284C | MRPL10 | MRPL18 | 14 | 104102 | 103134 | C | Verified |
| YNL264C | PDR17 | SFH4|ISS1 | 14 | 146616 | 145564 | C | Verified |
| YNL263C | YIF1 |  | 14 | 147841 | 146897 | C | Verified |
| YNL258C | DSL1 |  | 14 | 160374 | 158110 | C | Verified |
| YNL257C | SIP3 |  | 14 | 164321 | 160632 | C | Verified |
| YNL255C | GIS2 |  | 14 | 167791 | 167330 | C | Verified |
| YNL252C | MRPL17 | MRPL30 | 14 | 172287 | 171442 | C | Verified |
| YNL251C | NRD1 |  | 14 | 174316 | 172589 | C | Verified |
| YNL249C | MPA43 |  | 14 | 181023 | 179395 | C | Verified |
| YNL231C | PDR16 | SFH3 | 14 | 217043 | 215988 | C | Verified |
| YNL229C | URE2 | [URE3] | 14 | 220202 | 219138 | C | Verified |
| YNL213C |  |  | 14 | 247105 | 246461 | C | Verified |
| YNL201C | PSY2 |  | 14 | 263204 | 260628 | C | Verified |
| YNL199C | GCR2 |  | 14 | 266531 | 264927 | C | Verified |
| YNL185C | MRPL19 |  | 14 | 292671 | 292195 | C | Verified |
| YNL183C | NPR1 |  | 14 | 295511 | 293139 | C | Verified |
| YNL182C | IPI3 |  | 14 | 297629 | 295962 | C | Verified |
| YNL177C | MRPL22 |  | 14 | 304617 | 303688 | C | Verified |
| YNL176C |  |  | 14 | 306982 | 305072 | C | Uncharacterized |
| YNL175C | NOP13 |  | 14 | 308614 | 307403 | C | Verified |
| YNL154C | YCK2 |  | 14 | 345278 | 343638 | C | Verified |
| YNL151C | RPC31 | RPC8|ACP2 | 14 | 348523 | 347768 | C | Verified |
| YNL149C | PGA2 |  | 14 | 349758 | 349369 | C | Uncharacterized |
| YNL144C |  |  | 14 | 355044 | 352822 | C | Uncharacterized |
| YNL139C | RLR1 | LDB5|ZRG13|THO2 | 14 | 365719 | 360926 | C | Verified |
| YNL118C | DCP2 | PSU1 | 14 | 405566 | 402654 | C | Verified |
| YNL111C | CYB5 |  | 14 | 417304 | 416942 | C | Verified |
| YNL110C | NOP15 |  | 14 | 418490 | 417828 | C | Verified |
| YNL084C | END3 |  | 14 | 471104 | 470055 | C | Verified |
| YNL081C | SWS2 |  | 14 | 476620 | 476189 | C | Verified |
| YNL080C | EOS1 |  | 14 | 478033 | 476933 | C | Verified |
| YNL079C | TPM1 |  | 14 | 479166 | 478567 | C | Verified |
| YNL064C | YDJ1 | HSP40|MAS5 | 14 | 507098 | 505869 | C | Verified |
| YNL058C |  |  | 14 | 516714 | 515764 | C | Uncharacterized |
| YNL049C | SFB2 |  | 14 | 537912 | 535282 | C | Verified |
| YNL037C | IDH1 |  | 14 | 559003 | 557921 | C | Verified |
| YNL031C | HHT2 |  | 14 | 576051 | 575641 | C | Verified |
| YNL029C | KTR5 |  | 14 | 578774 | 577206 | C | Verified |
| YNL025C | SSN8 | CycC|RYE2|UME3|SRB11|  NUT9|GIG3 | 14 | 585292 | 584321 | C | Verified |
| YNL023C | FAP1 |  | 14 | 591161 | 588264 | C | Verified |
| YNL022C |  |  | 14 | 592900 | 591428 | C | Uncharacterized |
| YNL020C | ARK1 |  | 14 | 597540 | 595624 | C | Verified |
| YNL005C | MRP7 | MRPL2 | 14 | 622430 | 621315 | C | Verified |
| YNR003C | RPC34 |  | 14 | 635299 | 634346 | C | Verified |
| YNR013C | PHO91 |  | 14 | 651714 | 649030 | C | Verified |
| YNR029C |  |  | 14 | 678490 | 677201 | C | Uncharacterized |
| YNR037C | RSM19 |  | 14 | 695329 | 695054 | C | Verified |
| YNR039C | ZRG17 |  | 14 | 699433 | 697616 | C | Verified |
| YNR041C | COQ2 |  | 14 | 701661 | 700543 | C | Verified |
| YNR049C | MSO1 |  | 14 | 713657 | 713025 | C | Verified |
| YNR051C | BRE5 |  | 14 | 718329 | 716782 | C | Verified |
| YNR052C | POP2 | CAF1 | 14 | 720649 | 719348 | C | Verified |
| YNR055C | HOL1 |  | 14 | 730187 | 728427 | C | Verified |
| YNR061C |  |  | 14 | 743540 | 742881 | C | Uncharacterized |
| YNR067C | DSE4 | ENG1 | 14 | 759099 | 755746 | C | Verified |
| YOL158C | ENB1 | ARN4 | 15 | 21310 | 19490 | C | Verified |
| YOL147C | PEX11 | PMP27|PMP24 | 15 | 48642 | 47932 | C | Verified |
| YOL139C | CDC33 | TIF45 | 15 | 61024 | 60383 | C | Verified |
| YOL128C | YGK3 |  | 15 | 79479 | 78352 | C | Verified |
| YOL124C | TRM11 |  | 15 | 86757 | 85456 | C | Verified |
| YOL120C | RPL18A | RP28A | 15 | 94402 | 93395 | C | Verified |
| YOL119C | MCH4 |  | 15 | 96361 | 94856 | C | Verified |
| YOL118C |  |  | 15 | 96916 | 96608 | C | Dubious |
| YOL099C |  |  | 15 | 132508 | 132017 | C | Dubious |
| YOL098C |  |  | 15 | 135838 | 132725 | C | Uncharacterized |
| YOL097C | WRS1 | HRE342 | 15 | 137825 | 136527 | C | Verified |
| YOL087C |  |  | 15 | 158637 | 155287 | C | Uncharacterized |
| YOL086C | ADH1 | ADC1 | 15 | 160594 | 159548 | C | Verified |
| YOL077C | BRX1 |  | 15 | 186723 | 185848 | C | Verified |
| YOL075C |  |  | 15 | 193542 | 189658 | C | Uncharacterized |
| YOL068C | HST1 |  | 15 | 201879 | 200368 | C | Verified |
| YOL066C | RIB2 | PUS8 | 15 | 204471 | 202696 | C | Verified |
| YOL065C | INP54 |  | 15 | 205885 | 204731 | C | Verified |
| YOL063C | CRT10 | HUS1 | 15 | 210265 | 207392 | C | Verified |
| YOL052C | SPE2 |  | 15 | 233635 | 232445 | C | Verified |
| YOL043C | NTG2 | SCR2 | 15 | 249533 | 248391 | C | Verified |
| YOL040C | RPS15 | RPS21 | 15 | 253576 | 253148 | C | Verified |
| YOL027C | MDM38 | MKH1 | 15 | 273725 | 272004 | C | Verified |
| YOL026C | MIM1 | TOM13 | 15 | 274354 | 274013 | C | Verified |
| YOR006C |  |  | 15 | 338622 | 337681 | C | Uncharacterized |
| YOR021C |  |  | 15 | 373439 | 372798 | C | Uncharacterized |
| YOR023C | AHC1 |  | 15 | 377712 | 376012 | C | Verified |
| YOR035C | SHE4 | DIM1 | 15 | 400104 | 397735 | C | Verified |
| YOR051C |  | YOR29-02 | 15 | 426086 | 424848 | C | Uncharacterized |
| YOR064C | YNG1 | YOR29-15 | 15 | 446740 | 446081 | C | Verified |
| YOR070C | GYP1 | YOR29-21 | 15 | 457822 | 455909 | C | Verified |
| YOR074C | CDC21 | YOR29-25|TMP1|CRT9 | 15 | 467591 | 466677 | C | Verified |
| YOR076C | SKI7 | YOR29-27 | 15 | 471622 | 469379 | C | Verified |
| YOR090C | PTC5 | PPP1 | 15 | 492843 | 491125 | C | Verified |
| YOR095C | RKI1 |  | 15 | 504329 | 503553 | C | Verified |
| YOR098C | NUP1 |  | 15 | 511179 | 507949 | C | Verified |
| YOR103C | OST2 |  | 15 | 516842 | 516450 | C | Verified |
| YOR138C | RUP1 |  | 15 | 586325 | 584310 | C | Verified |
| YOR141C | ARP8 |  | 15 | 592588 | 589943 | C | Verified |
| YOR145C | PNO1 | RRP20|DIM2 | 15 | 606172 | 605348 | C | Verified |
| YOR164C |  |  | 15 | 644333 | 643395 | C | Verified |
| YOR167C | RPS28A | RPS33A | 15 | 649008 | 648805 | C | Verified |
| YOR180C | DCI1 | ECI2 | 15 | 675168 | 674353 | C | Verified |
| YOR201C | MRM1 | PET56 | 15 | 721709 | 720471 | C | Verified |
| YOR207C | RET1 | RPC128|RPC2|PDS2 | 15 | 733458 | 730009 | C | Verified |
| YOR213C | SAS5 |  | 15 | 745282 | 744536 | C | Verified |
| YOR245C | DGA1 |  | 15 | 795333 | 794077 | C | Verified |
| YOR246C |  |  | 15 | 796794 | 795802 | C | Uncharacterized |
| YOR259C | RPT4 | SUG2|PCS1|CRL13 | 15 | 813708 | 812395 | C | Verified |
| YOR261C | RPN8 |  | 15 | 816931 | 815915 | C | Verified |
| YOR270C | VPH1 |  | 15 | 830573 | 828051 | C | Verified |
| YOR275C | RIM20 |  | 15 | 841068 | 839083 | C | Verified |
| YOR281C | PLP2 |  | 15 | 847128 | 846268 | C | Verified |
| YOR306C | MCH5 |  | 15 | 891430 | 889865 | C | Verified |
| YOR307C | SLY41 |  | 15 | 894089 | 892728 | C | Verified |
| YOR311C | HSD1 |  | 15 | 899925 | 899053 | C | Verified |
| YOR312C | RPL20B | RPL18A1 | 15 | 901191 | 900247 | C | Verified |
| YOR320C | GNT1 |  | 15 | 915091 | 913616 | C | Verified |
| YOR322C | LDB19 |  | 15 | 921059 | 918603 | C | Verified |
| YOR323C | PRO2 |  | 15 | 922902 | 921532 | C | Verified |
| YOR324C | FRT1 | HPH1 | 15 | 925037 | 923229 | C | Verified |
| YOR329C | SCD5 | FTB1 | 15 | 939346 | 936728 | C | Verified |
| YOR330C | MIP1 |  | 15 | 943382 | 939618 | C | Verified |
| YOR340C | RPA43 |  | 15 | 960179 | 959199 | C | Verified |
| YOR357C | SNX3 | GRD19 | 15 | 1009710 | 1009222 | C | Verified |
| YOR360C | PDE2 | SRA5 | 15 | 1014819 | 1013239 | C | Verified |
| YOR363C | PIP2 | OAF2 | 15 | 1023210 | 1020220 | C | Verified |
| YOR370C | MRS6 | MSI4 | 15 | 1030992 | 1029181 | C | Verified |
| YPL279C |  |  | 16 | 14355 | 13228 | C | Uncharacterized |
| YPL263C | KEL3 |  | 16 | 46506 | 44551 | C | Verified |
| YPL258C | THI21 |  | 16 | 55153 | 53498 | C | Verified |
| YPL253C | VIK1 |  | 16 | 73006 | 71063 | C | Verified |
| YPL244C | HUT1 |  | 16 | 88033 | 87014 | C | Verified |
| YPL236C |  |  | 16 | 102702 | 101608 | C | Uncharacterized |
| YPL234C | TFP3 | VMA11|CLS9 | 16 | 105440 | 104946 | C | Verified |
| YPL212C | PUS1 |  | 16 | 153148 | 151514 | C | Verified |
| YPL202C | AFT2 |  | 16 | 169337 | 168087 | C | Verified |
| YPL199C |  |  | 16 | 172754 | 172032 | C | Uncharacterized |
| YPL184C |  |  | 16 | 197787 | 195949 | C | Uncharacterized |
| YPL174C | NIP100 | PAC13 | 16 | 222772 | 220166 | C | Verified |
| YPL162C |  |  | 16 | 244026 | 243205 | C | Uncharacterized |
| YPL161C | BEM4 | ROM7 | 16 | 246219 | 244318 | C | Verified |
| YPL158C |  |  | 16 | 254309 | 252033 | C | Uncharacterized |
| YPL138C | SPP1 | SAF41|CPS40 | 16 | 292426 | 291365 | C | Verified |
| YPL127C | HHO1 |  | 16 | 309603 | 308827 | C | Verified |
| YPL110C | GDE1 |  | 16 | 344738 | 341067 | C | Verified |
| YPL106C | SSE1 | MSI3|LPG3 | 16 | 352272 | 350191 | C | Verified |
| YPL103C |  |  | 16 | 359403 | 357997 | C | Uncharacterized |
| YPL094C | SEC62 | LPG14 | 16 | 370660 | 369836 | C | Verified |
| YPL089C | RLM1 |  | 16 | 381147 | 379117 | C | Verified |
| YPL071C |  |  | 16 | 420515 | 420045 | C | Uncharacterized |
| YPL069C | BTS1 |  | 16 | 423889 | 422882 | C | Verified |
| YPL068C |  |  | 16 | 425093 | 424212 | C | Uncharacterized |
| YPL049C | DIG1 | RST1 | 16 | 463836 | 462478 | C | Verified |
| YPL037C | EGD1 |  | 16 | 481898 | 481425 | C | Verified |
| YPL032C | SVL3 |  | 16 | 491362 | 488885 | C | Verified |
| YPL031C | PHO85 | LDB15 | 16 | 493035 | 492016 | C | Verified |
| YPL023C | MET12 |  | 16 | 506310 | 504337 | C | Verified |
| YPL015C | HST2 |  | 16 | 526880 | 525807 | C | Verified |
| YPR003C |  |  | 16 | 563765 | 561501 | C | Uncharacterized |
| YPR010C | RPA135 | SRP3|RRN2|RPA2 | 16 | 581193 | 577582 | C | Verified |
| YPR023C | EAF3 |  | 16 | 610028 | 608823 | C | Verified |
| YPR060C | ARO7 | TYR7|OSM2|HGS1 | 16 | 675628 | 674858 | C | Verified |
| YPR073C | LTP1 |  | 16 | 692415 | 691930 | C | Verified |
| YPR074C | TKL1 |  | 16 | 694835 | 692793 | C | Verified |
| YPR075C | OPY2 |  | 16 | 696816 | 695734 | C | Verified |
| YPR085C |  |  | 16 | 709826 | 708495 | C | Uncharacterized |
| YPR144C | NOC4 | UTP19 | 16 | 821419 | 819761 | C | Verified |
| YPR147C |  |  | 16 | 826555 | 825641 | C | Uncharacterized |
| YPR148C |  |  | 16 | 828136 | 826829 | C | Verified |
| YPR161C | SGV1 | BUR1 | 16 | 866418 | 864445 | C | Verified |
| YPR162C | ORC4 |  | 16 | 868300 | 866711 | C | Verified |
| YPR173C | VPS4 | DID6|VPT10|VPL4|GRD13|  END13|CSC1 | 16 | 887833 | 886520 | C | Verified |
| YPR179C | HDA3 | PLO1 | 16 | 895760 | 893793 | C | Verified |
| YPR190C | RPC82 | RPC3 | 16 | 919037 | 917073 | C | Verified |
| YBL106C | SRO77 | SNI2|SOP2 | 2 | 13879 | 10847 | C | Verified |
| YBL103C | RTG3 |  | 2 | 23535 | 22075 | C | Verified |
| YBL091C | MAP2 |  | 2 | 48625 | 47360 | C | Verified |
| YBL088C | TEL1 |  | 2 | 59379 | 51016 | C | Verified |
| YBL080C | PET112 |  | 2 | 74692 | 73067 | C | Verified |
| YBL061C | SKT5 | CSD4|CHS4|CAL2 | 2 | 107408 | 105318 | C | Verified |
| YBL055C |  | Tat-D | 2 | 116832 | 115576 | C | Verified |
| YBL042C | FUI1 |  | 2 | 140263 | 138344 | C | Verified |
| YBL039C | URA7 |  | 2 | 145731 | 143992 | C | Verified |
| YBL036C |  |  | 2 | 151223 | 150450 | C | Verified |
| YBL035C | POL12 |  | 2 | 153613 | 151496 | C | Verified |
| YBL023C | MCM2 |  | 2 | 177529 | 174923 | C | Verified |
| YBL007C | SLA1 |  | 2 | 216369 | 212635 | C | Verified |
| YBR007C | DSF2 |  | 2 | 251017 | 248807 | C | Uncharacterized |
| YBR008C | FLR1 |  | 2 | 254210 | 252564 | C | Verified |
| YBR023C | CHS3 | KTI2|DIT101|CSD2|CAL1 | 2 | 287925 | 284428 | C | Verified |
| YBR025C | OLA1 |  | 2 | 291865 | 290681 | C | Verified |
| YBR049C | REB1 | GRF2 | 2 | 336816 | 334384 | C | Verified |
| YBR057C | MUM2 | SPOT8 | 2 | 353291 | 352191 | C | Verified |
| YBR058C | UBP14 | GID6 | 2 | 356015 | 353670 | C | Verified |
| YBR058C-A | TSC3 |  | 2 | 356564 | 356322 | C | Verified |
| YBR067C | TIP1 |  | 2 | 372732 | 372100 | C | Verified |
| YBR069C | TAT1 | VAP1 | 2 | 378430 | 376571 | C | Verified |
| YBR070C | ALG14 |  | 2 | 379931 | 379218 | C | Verified |
| YBR072C-A |  |  | 2 | 383016 | 382855 | C | Uncharacterized |
| YBR079C | RPG1 | TIF32 | 2 | 398271 | 395377 | C | Verified |
| YBR080C | SEC18 | ANU4 | 2 | 400884 | 398608 | C | Verified |
| YBR090C |  |  | 2 | 427052 | 426327 | C | Uncharacterized |
| YBR091C | MRS5 | TIM12 | 2 | 427478 | 427149 | C | Verified |
| YBR092C | PHO3 |  | 2 | 429095 | 427692 | C | Verified |
| YBR095C | RXT2 | RAF60 | 2 | 435692 | 434400 | C | Verified |
| YBR105C | VID24 | GID4 | 2 | 451963 | 450875 | C | Verified |
| YBR107C | IML3 | MCM19 | 2 | 454524 | 453787 | C | Verified |
| YBR109C | CMD1 | CaM | 2 | 458356 | 457913 | C | Verified |
| YBR111C | YSA1 | RMA2 | 2 | 461867 | 461172 | C | Verified |
| YBR123C | TFC1 |  | 2 | 486685 | 484736 | C | Verified |
| YBR129C | OPY1 |  | 2 | 495333 | 494347 | C | Verified |
| YBR130C | SHE3 |  | 2 | 496863 | 495586 | C | Verified |
| YBR133C | HSL7 |  | 2 | 504281 | 501798 | C | Verified |
| YBR143C | SUP45 | SUP47|SUP1|SAL4 | 2 | 532176 | 530863 | C | Verified |
| YBR154C | RPB5 |  | 2 | 549003 | 548356 | C | Verified |
| YBR156C | SLI15 |  | 2 | 553194 | 551098 | C | Verified |
| YBR170C | NPL4 | HRD4 | 2 | 578081 | 576339 | C | Verified |
| YBR172C | SMY2 |  | 2 | 581367 | 579145 | C | Verified |
| YBR181C | RPS6B | RPS102|RPS101|LPG18 | 2 | 592769 | 591707 | C | Verified |
| YBR193C | MED8 |  | 2 | 609748 | 609077 | C | Verified |
| YBR198C | TAF5 | TAF90 | 2 | 618518 | 616122 | C | Verified |
| YBR204C |  |  | 2 | 633376 | 632249 | C | Uncharacterized |
| YBR216C | YBP1 |  | 2 | 657595 | 655571 | C | Verified |
| YBR218C | PYC2 |  | 2 | 662244 | 658702 | C | Verified |
| YBR221C | PDB1 |  | 2 | 666248 | 665148 | C | Verified |
| YBR222C | PCS60 | FAT2 | 2 | 668346 | 666715 | C | Verified |
| YBR229C | ROT2 | GLS2 | 2 | 679216 | 676352 | C | Verified |
| YBR231C | SWC5 | AOR1 | 2 | 683085 | 682174 | C | Verified |
| YBR234C | ARC40 |  | 2 | 686587 | 685433 | C | Verified |
| YBR236C | ABD1 |  | 2 | 691688 | 690378 | C | Verified |
| YBR238C |  |  | 2 | 697297 | 695102 | C | Verified |
| YBR239C |  |  | 2 | 699938 | 698349 | C | Uncharacterized |
| YBR243C | ALG7 | TUR1 | 2 | 706788 | 705442 | C | Verified |
| YBR245C | ISW1 | SGN2 | 2 | 711534 | 708145 | C | Verified |
| YBR260C | RGD1 |  | 2 | 734634 | 732634 | C | Verified |
| YBR269C |  |  | 2 | 742571 | 742155 | C | Uncharacterized |
| YBR272C | HSM3 |  | 2 | 747798 | 746356 | C | Verified |
| YBR283C | SSH1 |  | 2 | 770411 | 768939 | C | Verified |
| YBR288C | APM3 | YKS6 | 2 | 779459 | 778008 | C | Verified |
| YCL073C |  |  | 3 | 8326 | 6479 | C | Uncharacterized |
| YCL047C |  |  | 3 | 44437 | 43661 | C | Uncharacterized |
| YCL033C |  | MSRB | 3 | 63282 | 62776 | C | Uncharacterized |
| YCL031C | RRP7 |  | 3 | 65568 | 64675 | C | Verified |
| YCL011C | GBP2 | RLF6 | 3 | 103358 | 102075 | C | Verified |
| YCL002C |  |  | 3 | 111674 | 110843 | C | Uncharacterized |
| YCR002C | CDC10 |  | 3 | 118346 | 117378 | C | Verified |
| YCR009C | RVS161 | SPE161|FUS7|END6 | 3 | 131540 | 130743 | C | Verified |
| YCR017C | CWH43 |  | 3 | 147632 | 144771 | C | Verified |
| YCR018C | SRD1 |  | 3 | 148901 | 148236 | C | Verified |
| YCR028C | FEN2 |  | 3 | 172420 | 170882 | C | Verified |
| YCR028C-A | RIM1 |  | 3 | 173436 | 172946 | C | Verified |
| YCR038C | BUD5 |  | 3 | 199545 | 197617 | C | Verified |
| YCR043C |  |  | 3 | 206640 | 206257 | C | Uncharacterized |
| YCR045C |  |  | 3 | 209606 | 208131 | C | Uncharacterized |
| YCR046C | IMG1 |  | 3 | 210419 | 209910 | C | Verified |
| YCR047C | BUD23 |  | 3 | 211541 | 210714 | C | Verified |
| YCR054C | CTR86 |  | 3 | 220063 | 218372 | C | Verified |
| YCR064C |  |  | 3 | 228501 | 228091 | C | Dubious |
| YCR067C | SED4 |  | 3 | 236318 | 233121 | C | Verified |
| YCR090C |  |  | 3 | 272860 | 272312 | C | Uncharacterized |
| YCR095C |  |  | 3 | 289255 | 288167 | C | Uncharacterized |
| YDL231C | BRE4 |  | 4 | 42245 | 38868 | C | Verified |
| YDL226C | GCS1 |  | 4 | 52174 | 51116 | C | Verified |
| YDL224C | WHI4 |  | 4 | 56347 | 54398 | C | Verified |
| YDL223C | HBT1 |  | 4 | 60406 | 57266 | C | Verified |
| YDL217C | TIM22 |  | 4 | 68607 | 67984 | C | Verified |
| YDL216C | RRI1 | JAB1|CSN5 | 4 | 70320 | 68998 | C | Verified |
| YDL213C | NOP6 |  | 4 | 77967 | 77290 | C | Verified |
| YDL190C | UFD2 |  | 4 | 121593 | 118708 | C | Verified |
| YDL160C | DHH1 |  | 4 | 171931 | 170411 | C | Verified |
| YDL148C | NOP14 | UTP2 | 4 | 190587 | 188155 | C | Verified |
| YDL140C | RPO21 | SUA8|RPB220|RPB1 | 4 | 210562 | 205361 | C | Verified |
| YDL126C | CDC48 |  | 4 | 238664 | 236157 | C | Verified |
| YDL125C | HNT1 |  | 4 | 239606 | 239019 | C | Verified |
| YDL119C |  |  | 4 | 247612 | 246689 | C | Uncharacterized |
| YDL113C | ATG20 | CVT20|SNX42 | 4 | 258555 | 256633 | C | Verified |
| YDL111C | RRP42 |  | 4 | 264110 | 263313 | C | Verified |
| YDL106C | PHO2 | GRF10|BAS2 | 4 | 271901 | 270222 | C | Verified |
| YDL101C | DUN1 |  | 4 | 281848 | 280307 | C | Verified |
| YDL098C | SNU23 |  | 4 | 285165 | 284581 | C | Verified |
| YDL097C | RPN6 | NAS4 | 4 | 286695 | 285391 | C | Verified |
| YDL091C | UBX3 |  | 4 | 294759 | 293392 | C | Verified |
| YDL090C | RAM1 | STE16|SGP2|SCG2|FUS8|  DPR1 | 4 | 296329 | 295034 | C | Verified |
| YDL086C-A |  |  | 4 | 302089 | 301655 | C | Dubious |
| YDL081C | RPP1A | RPLA1 | 4 | 310122 | 309802 | C | Verified |
| YDL080C | THI3 | KID1 | 4 | 312471 | 310642 | C | Verified |
| YDL078C | MDH3 |  | 4 | 316388 | 315357 | C | Verified |
| YDL063C |  |  | 4 | 340134 | 338272 | C | Uncharacterized |
| YDL055C | PSA1 | SRB1|VIG9|MPG1 | 4 | 356759 | 355674 | C | Verified |
| YDL053C | PBP4 |  | 4 | 362256 | 361699 | C | Verified |
| YDL052C | SLC1 |  | 4 | 363583 | 362672 | C | Verified |
| YDL045C | FAD1 |  | 4 | 373605 | 372685 | C | Verified |
| YDL044C | MTF2 | NAM1 | 4 | 375286 | 373964 | C | Verified |
| YDL043C | PRP11 | RNA11 | 4 | 376477 | 375677 | C | Verified |
| YDL042C | SIR2 |  | 4 | 378442 | 376754 | C | Verified |
| YDL035C | GPR1 |  | 4 | 392054 | 389169 | C | Verified |
| YDL033C | SLM3 | MTU1|MTO2 | 4 | 393909 | 392656 | C | Verified |
| YDL018C | ERP3 |  | 4 | 423507 | 422830 | C | Verified |
| YDL005C | MED2 |  | 4 | 442306 | 441011 | C | Verified |
| YDL002C | NHP10 | HMO2 | 4 | 447575 | 446964 | C | Verified |
| YDR017C | KCS1 |  | 4 | 482264 | 479112 | C | Verified |
| YDR027C | VPS54 | TCS3|CGP1|LUV1 | 4 | 497315 | 494646 | C | Verified |
| YDR028C | REG1 | SRN1|SPP43|PZF240|HEX2 | 4 | 500876 | 497832 | C | Verified |
| YDR046C | BAP3 | PAP1 | 4 | 550574 | 548760 | C | Verified |
| YDR050C | TPI1 |  | 4 | 556470 | 555724 | C | Verified |
| YDR072C | IPT1 | KTI6|SYR4 | 4 | 591342 | 589759 | C | Verified |
| YDR084C | TVP23 |  | 4 | 614001 | 613402 | C | Verified |
| YDR090C |  |  | 4 | 625995 | 625063 | C | Uncharacterized |
| YDR098C | GRX3 |  | 4 | 645032 | 644175 | C | Verified |
| YDR111C | ALT2 |  | 4 | 679761 | 678238 | C | Uncharacterized |
| YDR117C | TMA64 | RBF64 | 4 | 685640 | 683943 | C | Verified |
| YDR129C | SAC6 | ABP67 | 4 | 715376 | 713337 | C | Verified |
| YDR130C | FIN1 |  | 4 | 716619 | 715744 | C | Verified |
| YDR135C | YCF1 |  | 4 | 727548 | 723001 | C | Verified |
| YDR141C | DOP1 |  | 4 | 739994 | 734898 | C | Verified |
| YDR143C | SAN1 |  | 4 | 743871 | 742039 | C | Verified |
| YDR144C | MKC7 | YPS2 | 4 | 746098 | 744308 | C | Verified |
| YDR154C |  |  | 4 | 768750 | 768400 | C | Dubious |
| YDR155C | CPR1 | CPH1|CYP1 | 4 | 768997 | 768509 | C | Verified |
| YDR164C | SEC1 |  | 4 | 784212 | 782038 | C | Verified |
| YDR166C | SEC5 |  | 4 | 789218 | 786303 | C | Verified |
| YDR169C | STB3 |  | 4 | 793886 | 792345 | C | Verified |
| YDR181C | SAS4 |  | 4 | 827352 | 825907 | C | Verified |
| YDR190C | RVB1 | TIP48|TIH1|TIP49A | 4 | 841992 | 840601 | C | Verified |
| YDR198C | RKM2 |  | 4 | 853969 | 852530 | C | Verified |
| YDR200C | VPS64 | FAR9 | 4 | 856090 | 854276 | C | Verified |
| YDR219C | MFB1 |  | 4 | 906848 | 905451 | C | Verified |
| YDR228C | PCF11 |  | 4 | 923802 | 921922 | C | Verified |
| YDR239C |  |  | 4 | 943416 | 941053 | C | Verified |
| YDR240C | SNU56 | MUD10 | 4 | 945148 | 943670 | C | Verified |
| YDR253C | MET32 |  | 4 | 964561 | 963986 | C | Verified |
| YDR260C | SWM1 |  | 4 | 977225 | 976713 | C | Verified |
| YDR264C | AKR1 |  | 4 | 998319 | 996025 | C | Verified |
| YDR267C | CIA1 |  | 4 | 1003498 | 1002506 | C | Verified |
| YDR284C | DPP1 | ZRG1 | 4 | 1031415 | 1030546 | C | Verified |
| YDR292C | SRP101 |  | 4 | 1045007 | 1043142 | C | Verified |
| YDR295C | HDA2 | PLO2 | 4 | 1054643 | 1052619 | C | Verified |
| YDR300C | PRO1 |  | 4 | 1062787 | 1061501 | C | Verified |
| YDR305C | HNT2 | APH1 | 4 | 1073484 | 1072742 | C | Verified |
| YDR306C |  |  | 4 | 1075167 | 1073731 | C | Uncharacterized |
| YDR313C | PIB1 |  | 4 | 1090076 | 1089216 | C | Verified |
| YDR324C | UTP4 |  | 4 | 1116760 | 1114430 | C | Verified |
| YDR328C | SKP1 | CBF3D|MGO1 | 4 | 1126013 | 1125429 | C | Verified |
| YDR333C |  |  | 4 | 1135426 | 1133255 | C | Uncharacterized |
| YDR345C | HXT3 |  | 4 | 1164654 | 1162951 | C | Verified |
| YDR348C |  |  | 4 | 1171819 | 1170320 | C | Uncharacterized |
| YDR349C | YPS7 |  | 4 | 1174170 | 1172380 | C | Verified |
| YDR359C | EAF1 | VID21 | 4 | 1194877 | 1191929 | C | Verified |
| YDR362C | TFC6 |  | 4 | 1198689 | 1196671 | C | Verified |
| YDR364C | CDC40 | SLU4|SLT15|PRP17 | 4 | 1204202 | 1202835 | C | Verified |
| YDR365C | ESF1 |  | 4 | 1206375 | 1204489 | C | Verified |
| YDR369C | XRS2 |  | 4 | 1217572 | 1215008 | C | Verified |
| YDR372C | VPS74 | API1 | 4 | 1222141 | 1221104 | C | Verified |
| YDR384C | ATO3 |  | 4 | 1242023 | 1241196 | C | Verified |
| YDR404C | RPB7 |  | 4 | 1277161 | 1276646 | C | Verified |
| YDR408C | ADE8 |  | 4 | 1288851 | 1288207 | C | Verified |
| YDR410C | STE14 |  | 4 | 1293083 | 1292364 | C | Verified |
| YDR411C | DFM1 |  | 4 | 1294386 | 1293361 | C | Verified |
| YDR422C | SIP1 |  | 4 | 1317765 | 1315318 | C | Verified |
| YDR423C | CAD1 | YAP2 | 4 | 1319267 | 1318038 | C | Verified |
| YDR424C | DYN2 | SLC1 | 4 | 1319833 | 1319379 | C | Verified |
| YDR428C |  |  | 4 | 1324231 | 1323446 | C | Uncharacterized |
| YDR429C | TIF35 |  | 4 | 1325293 | 1324469 | C | Verified |
| YDR430C | CYM1 | MOP112 | 4 | 1328462 | 1325493 | C | Verified |
| YDR449C | UTP6 |  | 4 | 1358894 | 1357572 | C | Verified |
| YDR451C | YHP1 |  | 4 | 1362173 | 1361112 | C | Verified |
| YDR454C | GUK1 | PUR5 | 4 | 1366819 | 1366256 | C | Verified |
| YDR461C-A |  |  | 4 | 1385758 | 1385516 | C | Uncharacterized |
| YDR470C | UGO1 |  | 4 | 1401206 | 1399698 | C | Verified |
| YDR479C | PEX29 |  | 4 | 1416866 | 1415202 | C | Verified |
| YDR485C | VPS72 | SWC2 | 4 | 1427199 | 1424812 | C | Verified |
| YDR486C | VPS60 | CHM5|MOS10 | 4 | 1428112 | 1427423 | C | Verified |
| YDR487C | RIB3 |  | 4 | 1428972 | 1428346 | C | Verified |
| YDR496C | PUF6 |  | 4 | 1443395 | 1441425 | C | Verified |
| YDR497C | ITR1 |  | 4 | 1445459 | 1443705 | C | Verified |
| YDR498C | SEC20 |  | 4 | 1446986 | 1445835 | C | Verified |
| YDR500C | RPL37B |  | 4 | 1450845 | 1450190 | C | Verified |
| YDR524C | AGE1 | SAT1 | 4 | 1488982 | 1487534 | C | Verified |
| YDR537C |  |  | 4 | 1511451 | 1510846 | C | Dubious |
| YDR541C |  |  | 4 | 1520682 | 1519648 | C | Uncharacterized |
| YEL064C | AVT2 |  | 5 | 31239 | 29797 | C | Verified |
| YEL063C | CAN1 |  | 5 | 33466 | 31694 | C | Verified |
| YEL055C | POL5 |  | 5 | 51539 | 48471 | C | Verified |
| YEL054C | RPL12A |  | 5 | 53218 | 52721 | C | Verified |
| YEL036C | ANP1 | MNN8|GEM3 | 5 | 84552 | 83050 | C | Verified |
| YEL034C-A |  |  | 5 | 86215 | 85613 | C | Dubious |
| YEL016C | NPP2 |  | 5 | 126218 | 124737 | C | Verified |
| YEL002C | WBP1 |  | 5 | 150013 | 148721 | C | Verified |
| YEL001C |  |  | 5 | 150977 | 150300 | C | Uncharacterized |
| YER003C | PMI40 | PMI | 5 | 159117 | 157735 | C | Verified |
| YER026C | CHO1 | PSS1 | 5 | 208473 | 207643 | C | Verified |
| YER027C | GAL83 | SPM1 | 5 | 210231 | 208978 | C | Verified |
| YER028C | MIG3 |  | 5 | 211875 | 210691 | C | Verified |
| YER029C | SMB1 |  | 5 | 213176 | 212586 | C | Verified |
| YER033C | ZRG8 |  | 5 | 221286 | 218056 | C | Verified |
| YER036C | ARB1 |  | 5 | 225198 | 223366 | C | Verified |
| YER048C | CAJ1 |  | 5 | 248156 | 246981 | C | Verified |
| YER052C | HOM3 | SIL4|BOR1 | 5 | 257957 | 256374 | C | Verified |
| YER053C | PIC2 |  | 5 | 259638 | 258736 | C | Verified |
| YER055C | HIS1 |  | 5 | 265784 | 264891 | C | Verified |
| YER056C | FCY2 | BRA7 | 5 | 268112 | 266511 | C | Verified |
| YER056C-A | RPL34A |  | 5 | 270183 | 269421 | C | Verified |
| YER057C | HMF1 | HIG1 | 5 | 271124 | 270735 | C | Verified |
| YER062C | HOR2 | GPP2 | 5 | 280680 | 279928 | C | Verified |
| YER064C |  |  | 5 | 284220 | 282703 | C | Uncharacterized |
| YER082C | UTP7 | KRE31 | 5 | 325932 | 324268 | C | Verified |
| YER083C | GET2 | RMD7|HUR2 | 5 | 327027 | 326170 | C | Verified |
| YER088C | DOT6 |  | 5 | 335184 | 333172 | C | Verified |
| YER094C | PUP3 | SCS32 | 5 | 349342 | 348725 | C | Verified |
| YER099C | PRS2 |  | 5 | 359057 | 358101 | C | Verified |
| YER105C | NUP157 |  | 5 | 372009 | 367834 | C | Verified |
| YER107C | GLE2 | RAE1 | 5 | 374541 | 373444 | C | Verified |
| YER109C | FLO8 | YER108C|PHD5 | 5 | 377610 | 375211 | C | Verified |
| YER110C | KAP123 | YRB4 | 5 | 382099 | 378758 | C | Verified |
| YER113C |  |  | 5 | 390048 | 387928 | C | Uncharacterized |
| YER114C | BOI2 | BEB1 | 5 | 393708 | 390586 | C | Verified |
| YER118C | SHO1 | SSU81 | 5 | 399051 | 397948 | C | Verified |
| YER119C | AVT6 |  | 5 | 400838 | 399492 | C | Verified |
| YER122C | GLO3 |  | 5 | 404348 | 402867 | C | Verified |
| YER124C | DSE1 |  | 5 | 409059 | 407338 | C | Verified |
| YER126C | NSA2 |  | 5 | 414175 | 413390 | C | Verified |
| YER130C |  |  | 5 | 422442 | 421111 | C | Uncharacterized |
| YER144C | UBP5 |  | 5 | 460218 | 457801 | C | Verified |
| YER149C | PEA2 | PPF2|DFG9 | 5 | 467465 | 466203 | C | Verified |
| YER155C | BEM2 | TSL1|SUP9|IPL2 | 5 | 482843 | 476340 | C | Verified |
| YER161C | SPT2 | EXA1|SIN1 | 5 | 500343 | 499342 | C | Verified |
| YER163C |  |  | 5 | 503777 | 503079 | C | Uncharacterized |
| YER168C | CCA1 | TNT1 | 5 | 522664 | 521024 | C | Verified |
| YFL039C | ACT1 | ABY1|END7 | 6 | 54695 | 53260 | C | Verified |
| YFL038C | YPT1 | YP2 | 6 | 55985 | 55365 | C | Verified |
| YFL027C | GYP8 |  | 6 | 81910 | 80417 | C | Verified |
| YFL025C | BST1 | PER17 | 6 | 87232 | 84143 | C | Verified |
| YFR005C | SAD1 |  | 6 | 155868 | 154522 | C | Verified |
| YFR016C |  |  | 6 | 180735 | 177034 | C | Verified |
| YFR025C | HIS2 |  | 6 | 204738 | 203731 | C | Verified |
| YFR028C | CDC14 | OAF3 | 6 | 210056 | 208401 | C | Verified |
| YFR039C |  |  | 6 | 233531 | 231999 | C | Uncharacterized |
| YFR043C |  |  | 6 | 239814 | 239101 | C | Uncharacterized |
| YFR044C | DUG1 |  | 6 | 241424 | 239979 | C | Verified |
| YFR046C | CNN1 |  | 6 | 244146 | 243061 | C | Verified |
| YGL257C | MNT2 |  | 7 | 14157 | 12481 | C | Verified |
| YGL246C | RAI1 |  | 7 | 38780 | 37617 | C | Verified |
| YGL231C |  |  | 7 | 63620 | 63048 | C | Uncharacterized |
| YGL221C | NIF3 |  | 7 | 82291 | 81425 | C | Verified |
| YGL200C | EMP24 | BST2 | 7 | 123310 | 122699 | C | Verified |
| YGL199C |  |  | 7 | 124047 | 123577 | C | Dubious |
| YGL194C | HOS2 | RTL1 | 7 | 141732 | 140374 | C | Verified |
| YGL193C |  |  | 7 | 142233 | 141922 | C | Uncharacterized |
| YGL190C | CDC55 |  | 7 | 147395 | 145815 | C | Verified |
| YGL189C | RPS26A | RPS26 | 7 | 148594 | 148235 | C | Verified |
| YGL173C | KEM1 | XRN1|SKI1|SEP1|RAR5|DST2 | 7 | 180119 | 175533 | C | Verified |
| YGL161C | YIP5 |  | 7 | 200148 | 199216 | C | Verified |
| YGL146C |  |  | 7 | 229691 | 228756 | C | Uncharacterized |
| YGL143C | MRF1 |  | 7 | 235963 | 234722 | C | Verified |
| YGL127C | SOH1 | MED31 | 7 | 270781 | 270398 | C | Verified |
| YGL120C | PRP43 | JA1 | 7 | 283943 | 281640 | C | Verified |
| YGL107C | RMD9 |  | 7 | 306276 | 304336 | C | Verified |
| YGL104C | VPS73 |  | 7 | 310176 | 308716 | C | Verified |
| YGL094C | PAN2 |  | 7 | 334468 | 331121 | C | Verified |
| YGL084C | GUP1 |  | 7 | 352301 | 350619 | C | Verified |
| YGL075C | MPS2 | MMC1 | 7 | 368091 | 366928 | C | Verified |
| YGL069C |  | SRF3 | 7 | 375517 | 375053 | C | Dubious |
| YGL064C | MRH4 |  | 7 | 383211 | 381526 | C | Verified |
| YGL061C | DUO1 |  | 7 | 389714 | 388971 | C | Verified |
| YGL040C | HEM2 | SLU1 | 7 | 420561 | 419533 | C | Verified |
| YGL038C | OCH1 | LDB12|NGD29 | 7 | 426810 | 425368 | C | Verified |
| YGL037C | PNC1 |  | 7 | 427953 | 427303 | C | Verified |
| YGL035C | MIG1 | TDS22|SSN1|CAT4 | 7 | 433067 | 431553 | C | Verified |
| YGL034C |  |  | 7 | 433584 | 433219 | C | Dubious |
| YGL028C | SCW11 |  | 7 | 442914 | 441286 | C | Verified |
| YGL027C | CWH41 | DER7|GLS1 | 7 | 446148 | 443647 | C | Verified |
| YGL026C | TRP5 |  | 7 | 448540 | 446417 | C | Verified |
| YGL018C | JAC1 |  | 7 | 459669 | 459115 | C | Verified |
| YGL013C | PDR1 | TPE3|TPE1|TIL1|SMR2|NRA2|CYH3|BOR2|ANT1|AMY1 | 7 | 472303 | 469097 | C | Verified |
| YGL011C | SCL1 | PRC2 | 7 | 475252 | 474494 | C | Verified |
| YGL008C | PMA1 |  | 7 | 482671 | 479915 | C | Verified |
| YGL005C | COG7 | COD5 | 7 | 490551 | 489712 | C | Verified |
| YGL003C | CDH1 | HCT1 | 7 | 494178 | 492478 | C | Verified |
| YGR015C |  |  | 7 | 522080 | 521094 | C | Uncharacterized |
| YGR036C | CAX4 | CWH8 | 7 | 558872 | 558153 | C | Verified |
| YGR044C | RME1 | CSP1 | 7 | 583896 | 582994 | C | Verified |
| YGR062C | COX18 | OXA2 | 7 | 617282 | 616332 | C | Verified |
| YGR063C | SPT4 |  | 7 | 617828 | 617520 | C | Verified |
| YGR078C | PAC10 | RKS2|PFD3|GIM2 | 7 | 640375 | 639776 | C | Verified |
| YGR081C | SLX9 |  | 7 | 643784 | 643152 | C | Verified |
| YGR085C | RPL11B |  | 7 | 648911 | 648387 | C | Verified |
| YGR086C | PIL1 |  | 7 | 650621 | 649602 | C | Verified |
| YGR095C | RRP46 |  | 7 | 676346 | 675675 | C | Verified |
| YGR119C | NUP57 |  | 7 | 729676 | 728051 | C | Verified |
| YGR120C | COG2 | SEC35 | 7 | 730826 | 730038 | C | Verified |
| YGR123C | PPT1 |  | 7 | 738208 | 736667 | C | Verified |
| YGR130C |  |  | 7 | 753849 | 751399 | C | Uncharacterized |
| YGR147C | NAT2 |  | 7 | 786925 | 786059 | C | Verified |
| YGR159C | NSR1 | SHE5 | 7 | 807661 | 806417 | C | Verified |
| YGR168C |  |  | 7 | 834487 | 833357 | C | Uncharacterized |
| YGR171C | MSM1 |  | 7 | 842556 | 840829 | C | Verified |
| YGR172C | YIP1 |  | 7 | 843596 | 842850 | C | Verified |
| YGR177C | ATF2 |  | 7 | 850441 | 848834 | C | Verified |
| YGR178C | PBP1 | MRS16 | 7 | 853220 | 851052 | C | Verified |
| YGR179C | OKP1 |  | 7 | 854900 | 853680 | C | Verified |
| YGR184C | UBR1 | PTR1 | 7 | 865758 | 859906 | C | Verified |
| YGR192C | TDH3 | SSS2|HSP36|HSP35|GLD1 | 7 | 883815 | 882817 | C | Verified |
| YGR196C | FYV8 |  | 7 | 892191 | 889738 | C | Verified |
| YGR200C | ELP2 | TOT2 | 7 | 902275 | 899909 | C | Verified |
| YGR202C | PCT1 | CCT1|BSR2 | 7 | 904753 | 903479 | C | Verified |
| YGR207C |  |  | 7 | 911633 | 910848 | C | Verified |
| YGR210C |  |  | 7 | 914743 | 913508 | C | Uncharacterized |
| YGR231C | PHB2 |  | 7 | 953483 | 952551 | C | Verified |
| YGR239C | PEX21 |  | 7 | 970058 | 969192 | C | Verified |
| YGR240C | PFK1 |  | 7 | 973739 | 970776 | C | Verified |
| YGR240C-A |  |  | 7 | 974782 | 974582 | C | Uncharacterized |
| YGR241C | YAP1802 |  | 7 | 976586 | 974880 | C | Verified |
| YGR244C | LSC2 |  | 7 | 979324 | 978041 | C | Verified |
| YGR245C | SDA1 |  | 7 | 982073 | 979770 | C | Verified |
| YGR255C | COQ6 |  | 7 | 1003967 | 1002528 | C | Verified |
| YGR257C | MTM1 |  | 7 | 1007311 | 1006211 | C | Verified |
| YGR263C |  |  | 7 | 1019247 | 1017973 | C | Uncharacterized |
| YGR264C | MES1 | MESI | 7 | 1021859 | 1019604 | C | Verified |
| YGR274C | TAF1 | TAF145|TAF130 | 7 | 1043101 | 1039901 | C | Verified |
| YGR279C | SCW4 |  | 7 | 1049964 | 1048804 | C | Verified |
| YGR280C | PXR1 | GNO1 | 7 | 1051731 | 1050916 | C | Verified |
| YGR282C | BGL2 |  | 7 | 1058730 | 1057789 | C | Verified |
| YGR283C |  |  | 7 | 1060046 | 1059021 | C | Verified |
| YGR285C | ZUO1 |  | 7 | 1063159 | 1061858 | C | Verified |
| YHL035C | VMR1 |  | 8 | 32754 | 27976 | C | Verified |
| YHL033C | RPL8A | MAK7 | 8 | 36023 | 35253 | C | Verified |
| YHL032C | GUT1 |  | 8 | 38506 | 36377 | C | Verified |
| YHL031C | GOS1 |  | 8 | 39484 | 38813 | C | Verified |
| YHL023C | RMD11 |  | 8 | 62561 | 59121 | C | Verified |
| YHL016C | DUR3 |  | 8 | 74241 | 72034 | C | Verified |
| YHL011C | PRS3 |  | 8 | 81612 | 80650 | C | Verified |
| YHL010C |  |  | 8 | 83717 | 81960 | C | Uncharacterized |
| YHR003C |  |  | 8 | 111312 | 110023 | C | Verified |
| YHR005C | GPA1 | SCG1|DAC1|CDC70 | 8 | 114912 | 113494 | C | Verified |
| YHR005C-A | MRS11 | TIM10 | 8 | 115896 | 115615 | C | Verified |
| YHR007C | ERG11 | CYP51 | 8 | 121678 | 120086 | C | Verified |
| YHR007C-A |  |  | 8 | 122760 | 122545 | C | Uncharacterized |
| YHR009C |  |  | 8 | 125675 | 124104 | C | Uncharacterized |
| YHR013C | ARD1 |  | 8 | 131440 | 130724 | C | Verified |
| YHR016C | YSC84 | LSB4 | 8 | 138448 | 136874 | C | Verified |
| YHR019C | DED81 |  | 8 | 143551 | 141887 | C | Verified |
| YHR024C | MAS2 | MIF2 | 8 | 159185 | 157737 | C | Verified |
| YHR031C | RRM3 | RTT104 | 8 | 172963 | 170792 | C | Verified |
| YHR039C | MSC7 |  | 8 | 186802 | 184868 | C | Verified |
| YHR064C | SSZ1 | PDR13 | 8 | 227143 | 225527 | C | Verified |
| YHR065C | RRP3 |  | 8 | 229039 | 227534 | C | Verified |
| YHR077C | NMD2 | UPF2|SUA1|IFS1 | 8 | 255758 | 252376 | C | Verified |
| YHR082C | KSP1 |  | 8 | 271550 | 268461 | C | Verified |
| YHR100C |  |  | 8 | 314676 | 314119 | C | Uncharacterized |
| YHR115C | DMA1 | CHF1 | 8 | 341362 | 340112 | C | Verified |
| YHR144C | DCD1 |  | 8 | 388729 | 387791 | C | Verified |
| YHR147C | MRPL6 |  | 8 | 393286 | 392642 | C | Verified |
| YHR188C | GPI16 |  | 8 | 483837 | 482005 | C | Verified |
| YIL154C | IMP2' | IMP2 | 9 | 55021 | 53981 | C | Verified |
| YIL147C | SLN1 | YPD2 | 9 | 73453 | 69791 | C | Verified |
| YIL135C | VHS2 |  | 9 | 96375 | 95065 | C | Verified |
| YIL109C | SEC24 | ANU1 | 9 | 160162 | 157382 | C | Verified |
| YIL105C | SLM1 | LIT2 | 9 | 169638 | 167578 | C | Verified |
| YIL096C |  |  | 9 | 183124 | 182114 | C | Uncharacterized |
| YIL094C | LYS12 | LYS11|LYS10 | 9 | 187629 | 186514 | C | Verified |
| YIL085C | KTR7 |  | 9 | 202040 | 200487 | C | Verified |
| YIL069C | RPS24B | RPS24EB | 9 | 232366 | 231550 | C | Verified |
| YIL062C | ARC15 |  | 9 | 244459 | 243995 | C | Verified |
| YIL047C-A |  |  | 9 | 265466 | 265098 | C | Dubious |
| YIL047C | SYG1 |  | 9 | 267822 | 265114 | C | Verified |
| YIL043C | CBR1 | CBR5 | 9 | 274925 | 274071 | C | Verified |
| YIL038C | NOT3 |  | 9 | 282651 | 280141 | C | Verified |
| YIL035C | CKA1 |  | 9 | 288907 | 287789 | C | Verified |
| YIL034C | CAP2 |  | 9 | 290088 | 289225 | C | Verified |
| YIL033C | BCY1 | SRA1 | 9 | 291668 | 290418 | C | Verified |
| YIL009C-A | EST3 |  | 9 | 336209 | 335663 | C | Verified |
| YIL004C | BET1 | SLY12 | 9 | 348502 | 347943 | C | Verified |
| YIR008C | PRI1 |  | 9 | 374303 | 373074 | C | Verified |
| YIR031C | DAL7 | MSL2|MLS2 | 9 | 414676 | 413012 | C | Verified |
| YIR036C |  |  | 9 | 422862 | 422071 | C | Uncharacterized |
