# Supplementary figures and images for: Unphosphorylated SR-Like Protein Npl3 Stimulates RNA Polymerase II Elongation
Source: PLoS One. 2008 Sep 26;3(9):e3273. doi: 10.1371/journal.pone.0003273 (PMC2538588; doi:10.1371/journal.pone.0003273)

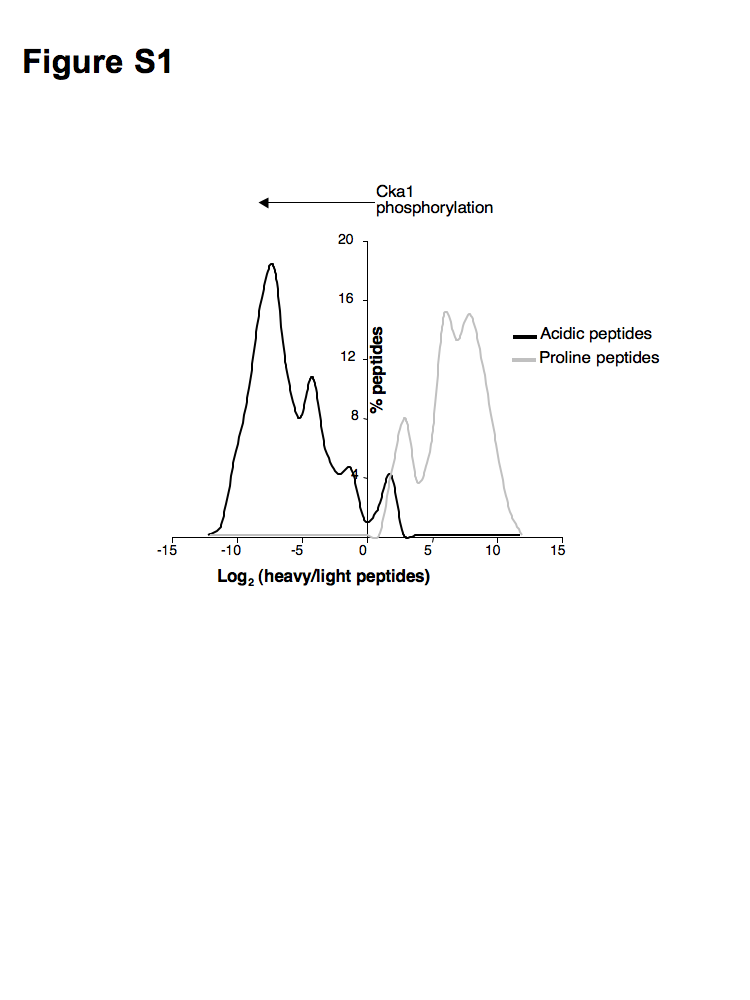

Supplement: Figure S1 — Distribution of the log2 ratios of the peptide libraries phosphorylated in vitro with Cka1 and measured by MS. Kinase assays utilizing peptide libraries representing acidic (L[LE]D[KDN]D[DA][LE][ST]D[EL]E[LEN][EL]K) and proline-directed ([KP]L[VKE]L[AP][NE][ST]P[KI][LKP]VV[KL]) motifs were performed. 20 µg of the non-phosphorylated libraries were used, and 0.2 µg of the heavy phosphorylated standard library was added upon reaction quenching with 0.15 % TFA. Reactions were desalted in a tC18 SepPak and enriched for phosphopeptides with PhosSelect IMAC resin (Sigma). Samples were desalted again prior to injection into a capillary (125 µm×18 cm) C18 column and analyzed by LC-MS/MS in a LTQ-Orbitrap mass spectrometer using a 60 minute-gradient and data dependent TOP10 method. MS/MS spectra were searched against a database containing the sequences for all the peptides in the libraries and all the sequences from E. coli protein sequence database (used for distraction purposes) in the forward and reverse directions. Results were filtered to <1% false positives and peptides were further quantified using the VistaQUANT algorithm. (0.07 MB TIF) [file pone.0003273.s003.tif]

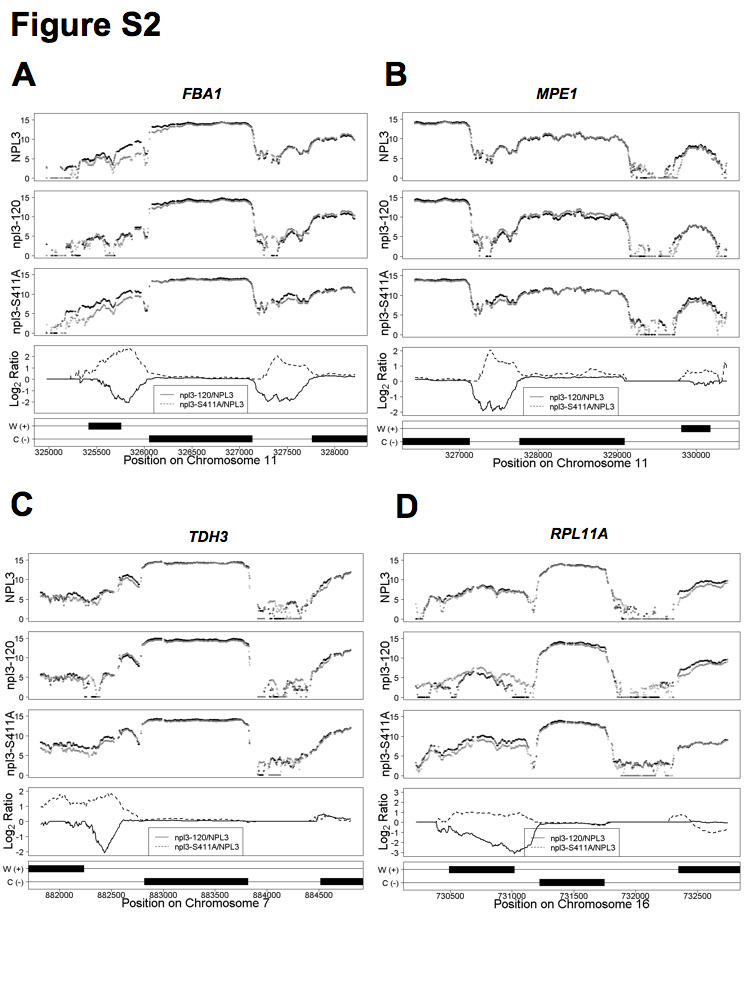

Supplement: Figure S2 — Opposing effects in termination demonstrated using RNA-binding or phosphorylation defective npl3 alleles. RNA was extracted from NPL3, npl3-120 and npl3-S411A and whole transcript sense strand cDNA was synthesized and used for hybridization to tiled arrays. Shown are the hybridization signals for twelve genes. The three top panels represent the individual expression intensities for each strain, with the black and gray dotted lines showing two independent experiments. The bottom panel shows the ratio of each mutant vs. wild-type; a solid line for npl3-120 vs. NPL3, or dashed line for npl3-S411A vs. NPL3, and the corresponding position and orientation (Watson (W+), shown for reference only, or Crick (C-) strand) of the genes (black solid bars). (0.17 MB TIF) [file pone.0003273.s004.tif]

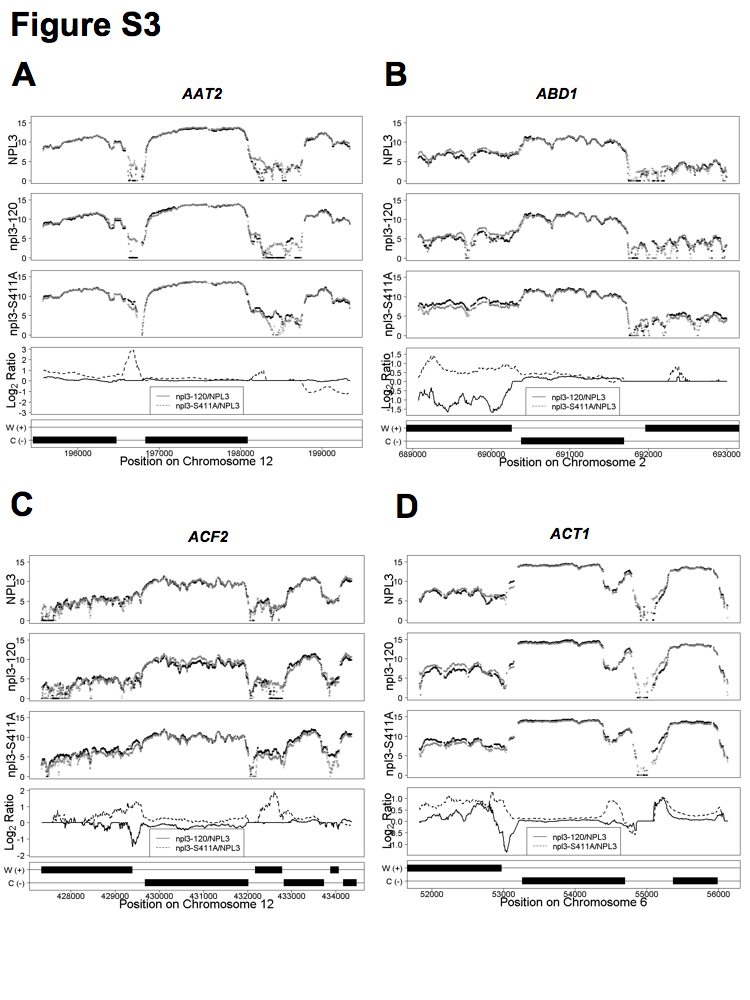

Supplement: Figure S3 — Opposing effects in termination demonstrated using RNA-binding or phosphorylation defective npl3 alleles. RNA was extracted from NPL3, npl3-120 and npl3-S411A and whole transcript sense strand cDNA was synthesized and used for hybridization to tiled arrays. Shown are the hybridization signals for twelve genes. The three top panels represent the individual expression intensities for each strain, with the black and gray dotted lines showing two independent experiments. The bottom panel shows the ratio of each mutant vs. wild-type; a solid line for npl3-120 vs. NPL3, or dashed line for npl3-S411A vs. NPL3, and the corresponding position and orientation (Watson (W+), shown for reference only, or Crick (C-) strand) of the genes (black solid bars). (0.18 MB TIF) [file pone.0003273.s005.tif]

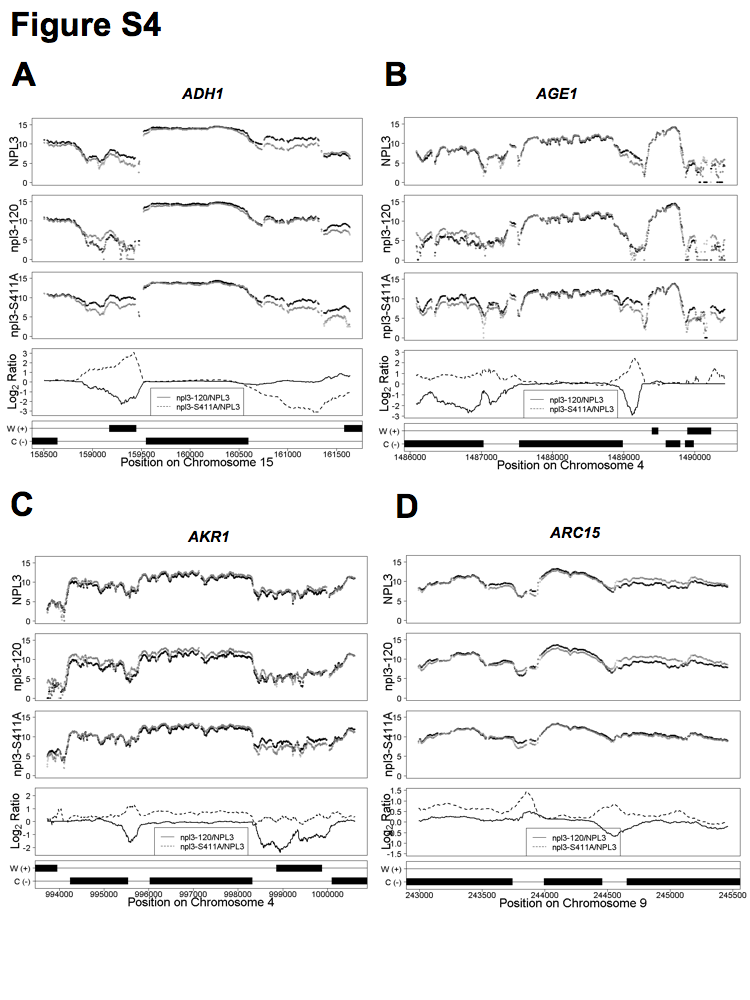

Supplement: Figure S4 — Opposing effects in termination demonstrated using RNA-binding or phosphorylation defective npl3 alleles. RNA was extracted from NPL3, npl3-120 and npl3-S411A and whole transcript sense strand cDNA was synthesized and used for hybridization to tiled arrays. Shown are the hybridization signals for twelve genes. The three top panels represent the individual expression intensities for each strain, with the black and gray dotted lines showing two independent experiments. The bottom panel shows the ratio of each mutant vs. wild-type; a solid line for npl3-120 vs. NPL3, or dashed line for npl3-S411A vs. NPL3, and the corresponding position and orientation (Watson (W+), shown for reference only, or Crick (C-) strand) of the genes (black solid bars). (0.17 MB TIF) [file pone.0003273.s006.tif]
